# Integrative network pharmacology, metabolomics and gut flora studies reveal mechanisms of action of *Rhododendron molle* (Blume) G. Don to ameliorate liver injury

**DOI:** 10.3389/fmicb.2025.1570229

**Published:** 2025-09-01

**Authors:** Xiaolei Jiang, Yafeng Zhuang, Tiancheng Meng, Tianwei Meng, Xinghua Li, Dan He, Hongyu Meng, Hong Chang

**Affiliations:** ^1^Department of Nephrology and Endocrinology, Dongzhimen Hospital, Beijing University of Chinese Medicine, Beijing, China; ^2^College of Pharmacy, Baotou Medical College, Baotou, China; ^3^College of Traditional Chinese Medicine, Beijing University of Chinese Medicine, Beijing, China; ^4^Graduate School, Heilongjiang University of Chinese Medicine, Harbin, China; ^5^Changzhi People’s Hospital Affiliated to Changzhi Medical College, Changzhi, China

**Keywords:** *Rhododendron molle* (Blume) G. Don, network pharmacology, molecular docking, metabolomics, intestinal flora

## Abstract

**Background:**

Liver injury (LI) is responsible for a significant number of fatalities each year. In the context of Mongolian medicine, *Rhododendron molle* (Blume) G. Don (RM) is utilized for its properties to treatment of hepatic disorders. However, the underlying mechanisms of its action remain poorly understood.

**Objectives:**

Clarifying the process through which RM enhances LI.

**Methods:**

The chemical constituents were subjected to analysis, and network pharmacology alongside molecular docking studies were conducted. Additionally, ELISA, staining techniques, metabolomic analyses, and 16S rDNA sequencing were performed.

**Results:**

A total of 17 components have been identified from RM, including liver disease-related compounds such as kaempferol, emodin, quercetin. Network pharmacology has identified notable genes that exhibit a strong binding affinity to active compounds, including emodin, which interacts with IL6 and PPARG, and aloeemodin, which binds to IL6 and AKT1. In a rat model of LI induced by CCL_4_, low dose (0.07875 g/kg) of RM demonstrated a reduction in ALT and γ-GT levels (*p* < 0.05). Metabolomic analysis indicated that RM has an impact on the concentrations of 13-OxoODE, morphine, and niacinamide in rat models exhibiting LI, simultaneously several metabolic pathways, including steroid biosynthesis, linoleic acid metabolism, and tryptophan metabolism. By integrating the findings from metabolomics with KEGG pathways, it was determined that RM may ameliorate LI by activating specific pathways and modulating fatty acid metabolic processes, particularly linoleic acid and arachidonic acid metabolism. Furthermore, low-dose RM (RML) was found to enhance beneficial gut microbiota such as *Lactobacillus*, suggesting its potential role in the regulation of intestinal homeostasis and barrier integrity.

**Conclusion:**

RML has the potential to enhance the composition of intestinal microbiota by through the differential regulation of various metabolized components, including 13-OxoODE, morphine, and niacinamide, it influences several metabolic pathways, notably steroid biosynthesis, lysine degradation, interconversions of pentose and glucuronate, as well as the metabolism of linoleic acid. Additionally, it may promote the proliferation of *HT002* and *Lactobacillus* probiotics, thereby contributing to the amelioration of LI. It establishes a robust foundation for future applications and the development of associated pharmaceuticals.

## 1 Introduction

Liver injury (LI) represents a significant global health issue, exhibiting a range of epidemiological characteristics that differ across regions and populations. A report published in 2023 indicates that chronic liver disease and LI rank among the foremost causes of mortality globally, resulting in an estimated 2 million deaths annually (Devarbhavi et al., 2023). The causes of LI are complex and varied, including factors such as viral infections and alcoholic liver disease (Hackstein et al., 2023), drug-induced hepatotoxicity (Zhang et al., 2024), and non-alcoholic fatty liver disease (Chen et al., 2024). These elements can initiate a series of pathological processes characterized by hepatic inflammation, fibrosis, and cirrhosis, which may eventually lead to liver failure or the development of hepatocellular carcinoma (Lin et al., 2023).

Under typical physiological conditions, the gut microbiota is involved in various physiological processes and is essential for the support and regulation of liver functions (Zhang et al., 2022). The disruption of microbial homeostasis may result in the impairment of the liver’s metabolic, detoxification, and immune regulatory functions (Zhang et al., 2024). The disruption of gut microbiota homeostasis is characterized by a decline in the population of beneficial bacterial taxa, including *Bacteroidota*, *Lactobacillus*, *Actinobacteriota*, *Proteobacteria*, and *Clostridium*. Concurrently, there is an overgrowth of pathogenic bacteria, such as *Erysipelotrichaceae_UCG-003*, *Lachnoclostridium*, *Enterococcus*, and *Dorea*. This dysbiosis further compromises the integrity of the intestinal barrier, leading to increased intestinal permeability. As a result, a greater number of bacteria and their metabolites can translocate into the bloodstream, thereby exacerbating the inflammatory response and the severity of intestinal inflammation (Li et al., 2022). Conversely, the immune disorder induced by LI will have an impact on the intestinal microecological environment, altering the habitat of gut microbiota. This disruption may inhibit the proliferation of beneficial bacteria while facilitating the growth of opportunistic pathogens (Zhang et al., 2024). Consequently, a detrimental cycle is established, perpetually advancing the progression and decline of LI.

The medicinal application of RM is derived from the desiccated flowers of *Rhododendron molle* (Guo et al., 2021a). It possesses properties that facilitate the expulsion of wind and the elimination of dampness, as well as the dispersion of blood stasis and the alleviation of pain. This treatment is frequently employed for conditions such as rheumatic arthralgia, unilateral or bilateral headaches, and swelling and pain resulting from trauma, in addition to persistent tinea (Chinese Pharmacopoeia Commission, 2020). “Bashaga” is a frequently utilized medicinal substance within the realm of Mongolian medicine, boasting a rich historical background and holding significant importance in this field. In clinical practice, this substance functions as a substitute for “Bashaga” medicinal herbs within the framework of Mongolian traditional medicine, specifically in certain areas of Hulunbuir City, Chifeng City, and Tongliao City in Inner Mongolia (Xiao et al., 2020). The “Compendium of Chinese Herbal Medicine-Mongolian Medicine Volume” documents that RM possesses therapeutic properties for the treatment of various conditions, including dermatological disorders, cardiovascular diseases, and hepatic diseases, among others.

Nevertheless, the rationale for its application and the mechanisms that underpin its therapeutic effects remain to be clarified (Zhao et al., 2017). Recent pharmacological investigations into RM have identified its various pharmacological properties, including anti-inflammatory, analgesic, anesthetic, hypotensive, insecticidal, antiviral, and anticancer activities (Cai et al., 2018; Guo et al., 2021b). The primary constituents of RM encompass diterpenoids, flavonoids, triterpenoids, and coumarins (Guo et al., 2020). Diterpenoids, including grayanotoxin I, rhodojaponin VI, and rhodojaponin V, are identified as the toxic constituents of RM. Administration of these compounds at elevated doses may result in neurotoxicity and cardiotoxicity, with the potential for fatal outcomes due to respiratory failure in extreme cases. Conversely, the administration of RM at lower doses has been associated with anti-inflammatory and analgesic properties (Liu et al., 2018). Recent research has indicated that the fingerprint of RM contains flavonoid constituents, including kaempferol and quercetin (Hu et al., 2024), both components play crucial roles within the paradigm of traditional Chinese medicine. They exhibit pharmacological properties that positively influence human health, including antioxidant, anti-inflammatory, anti-obesity, and hepatoprotective effects (Gu et al., 2023; Singh et al., 2023). In light of the therapeutic efficacy of “Bashaga”-type Mongolian medicines in the treatment of liver diseases observed in clinical practice, we aim to conduct further investigations into the mechanisms by which RM mitigates LI.

Consequently, this study aimed to investigate the ameliorative effects of RM on LI by employing a CCl_4_-induced LI model. Initially, we conducted a comprehensive analysis of the components of RM, followed by network pharmacology analysis and molecular docking of the identified constituents to establish a RM-target network and a “component-target-metabolite” network. This approach allowed for a thorough elucidation of the *in vivo* targets and signaling pathways associated with RM. Furthermore, we compared the pharmacodynamics of three different doses of RM through pathological assessments and ELISA, performed fecal metabolomics on both high and low dose groups, and analyzed the gut microbiota associated with the optimal dose. This study provides preliminary insights into the dosing of RM and investigates the mechanisms through which RM mitigates LI.

## 2 Result

### 2.1 Analysis of the components of RM

**Figure 1:**
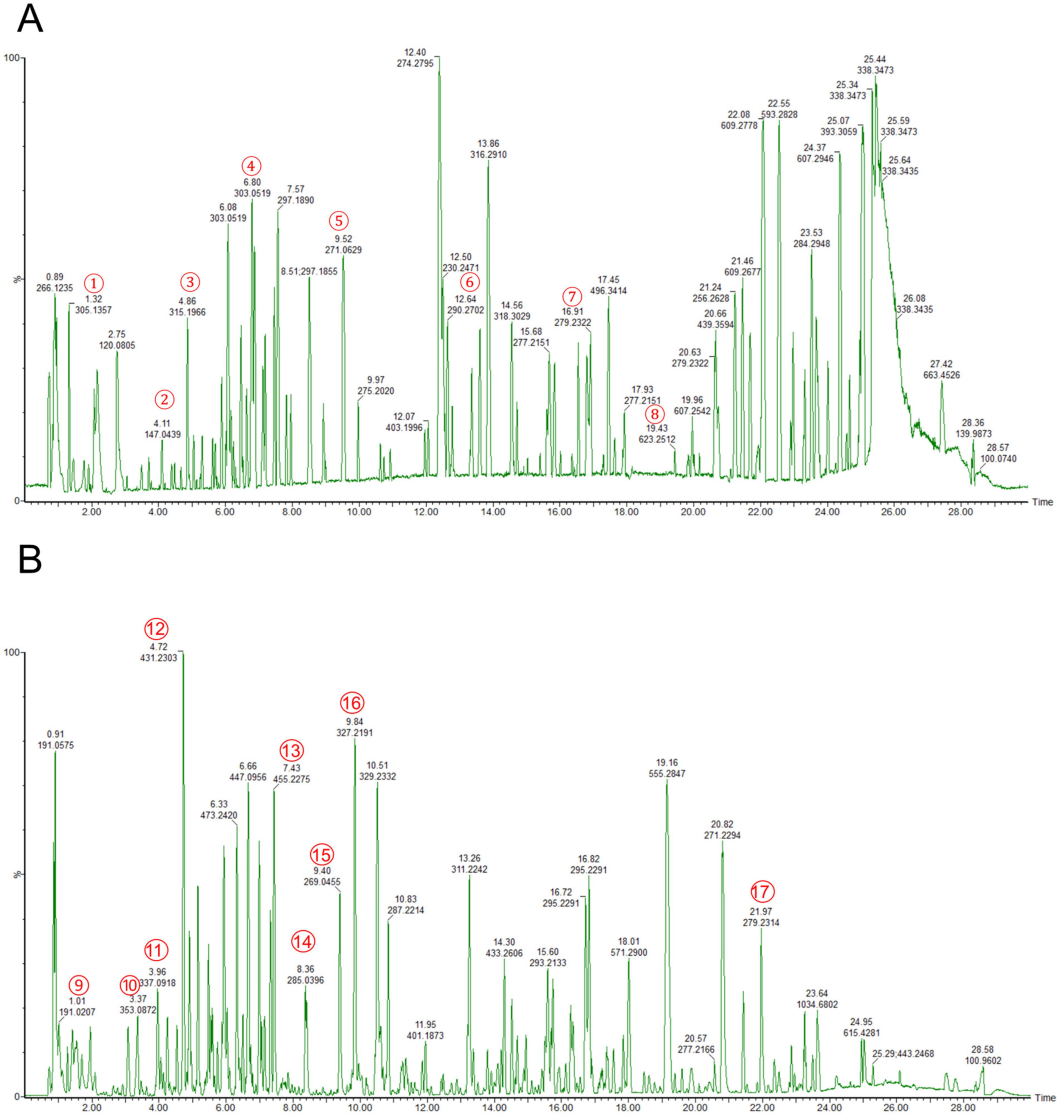
TIC of the components of RM in positive and negative ion modes. **(A)** TIC of the components of RM in positive ion mode. **(B)** TIC of the components of RM in negative ion mode. ① Resacetophenone. ② Benzylideneacetone. ③ Laccaic acid D. ④ Quercetin. ⑤ Emodin. ⑥ Adenosine. ⑦ Linolenic acid. ⑧ Sugiol. ⑨ Citric acid. ⑩ Heriguard. ⑪ 3-O-p-Coumaroylquinic acid. ⑫ Aloeemodin-Omega-O-beta-D-glucopyranoside. ⑬ Oleanic acid. ⑭ Kaempferol. ⑮ Aloeemodin. ⑯ Paeonoside. ⑰ Linoleic acid.

Total ion chromatograms of the RM were obtained in both negative and positive ion modes. An analysis of the mass spectrometric data, coupled with database comparisons and secondary identification methods, led to the identification of 17 components within the RM across both ionization modes (Figure 1 and Table 1). The identified components comprised citric acid, heriguard, oleanic acid (Wang et al., 2014), emodin, quercetin, kaempferol (Liu and Kong, 2009), among others.

**Table 1:** The active ingredient of RM identified by UPLC-Q/TOF-MS.

No.	Name	Formula	RT (min)	*m*/*z*	Mode	CAS	MS2
1	Resacetophenone	C_8_H_8_O_3_	1.32	305.10	POS	954-97-2	112.0489, 136.0618, 152.0557
2	Benzylideneacetone	C_10_H_10_O	4.11	315.13	POS	122-57-6	147.0803, 119.0855
3	Laccaic acid D	C_16_H_10_O_7_	4.86	315.05	POS	18499-84-8	269.1898, 279.1747, 297.1855
4	Quercetin	C_15_H_10_O_7_	6.80	303.05	POS	117-39-5	303.0159, 287.0556
5	Emodin	C_15_H_10_O_5_	9.52	271.06	POS	518-82-1	163.0359, 271.0606
6	Adenosine	C_10_H_13_N_5_O_4_	12.64	268.10	POS	58-61-7	136.0618, 119.0352, 137.0456
7	Linolenic acid	C_18_H_30_O_2_	16.91	279.23	POS	463-40-1	243.2113, 261.2218
8	Sugiol	C_20_H_28_O_2_	19.43	301.22	POS	511-05-7	139.1491, 273.1849, 123.1168
9	Citric acid	C_6_H_8_O_7_	1.01	191.02	NEG	77-92-9	111.0091, 173.0096, 191.0203
10	Heriguard	C_16_H_18_O_9_	3.37	353.09	NEG	327-97-9	191.0556, 353.0866, 351.0709
11	3-O-p-Coumaroylquinic acid	C_16_H_18_O_8_	3.96	337.09	NEG	5746-55-4	163, 337
12	Aloeemodin-Omega-O-beta-D-glucopyranoside	C_21_H_20_O_10_	4.72	431.10	NEG	50488-89-6	251.03398, 252.03604, 253.05132
13	Oleanic acid	C_30_H_48_O_3_	7.43	455.35	NEG	508-02-1	455.270700, 456.354300
14	Kaempferol	C_15_H_10_O_6_	8.36	285.04	NEG	520-18-3	285.0399, 286.0442, 117.0346
15	Aloeemodin	C_15_H_10_O_5_	9.40	269.04	NEG	481-72-1	117.03353, 149.02309, 269.04419
16	Paeonoside	C_15_H_20_O_8_	9.84	327.11	NEG	20309-70-0	309, 207, 291
17	Linoleic acid	C_18_H_32_O_2_	21.97	279.23	NEG	2197-37-7	279.2328, 280.2353

### 2.2 Network pharmacology

#### 2.2.1 Screening of targets of RM components

The 17 identified chemical constituents were systematically entered into the Swiss Target Prediction database, resulting in the identification of 16 potential active constituents, such as isorhamnetin, kaempferol, andromedotoxin along with their corresponding action targets. Subsequently, all targets underwent a screening and deduplication process, yielding a total of 370 unique constituent targets.

#### 2.2.2 Related targets of RM acting on LI

**Figure 2:**
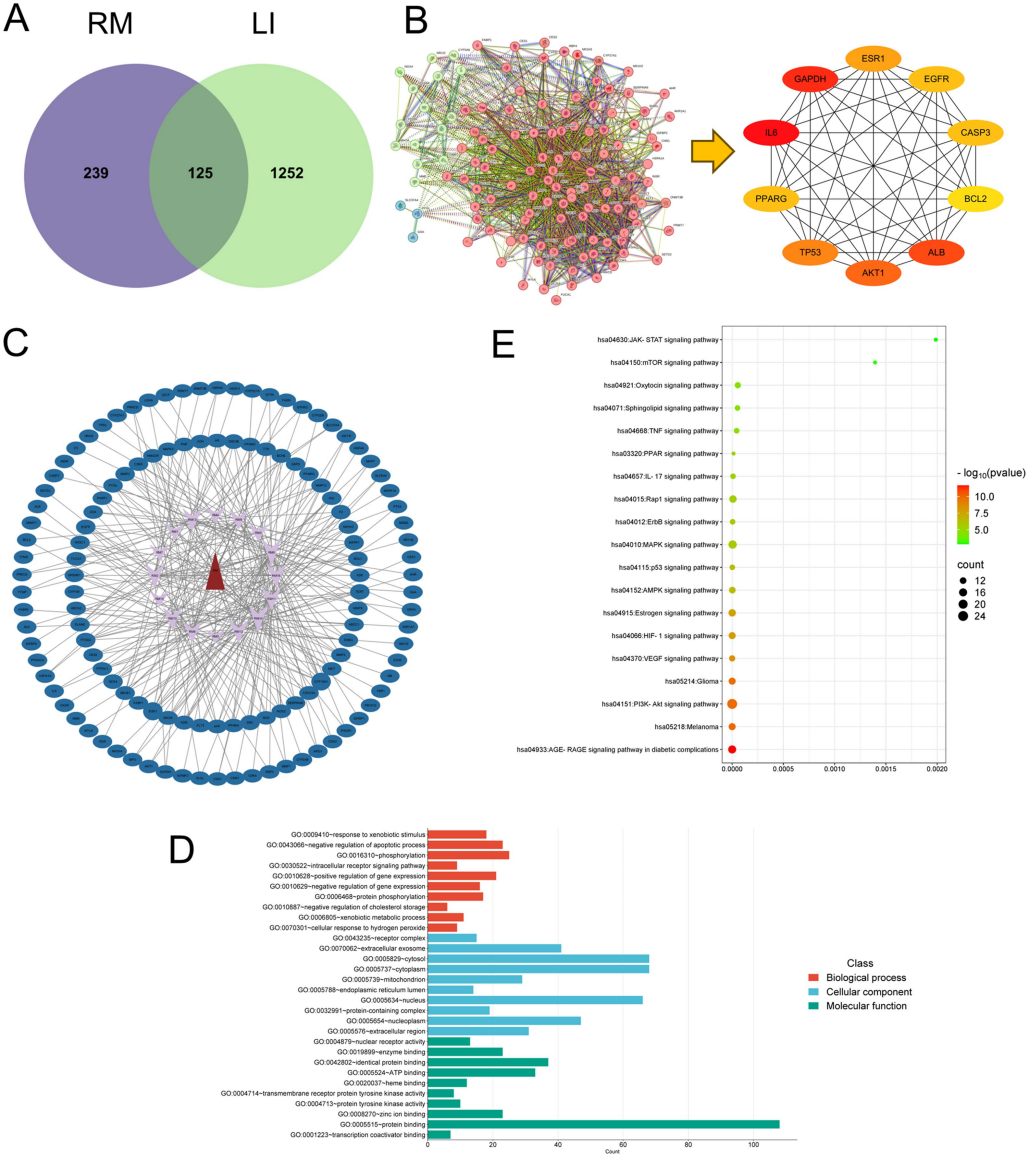
Network pharmacology research on the treatment of LI with RM. **(A)** The intersection targets between the potential active ingredients of RM and LI. **(B)** The core targets of RM for improving LI. **(C)** The “drug-ingredient-target” network. **(D)** GO enrichment analysis. **(E)** KEGG enrichment analysis.

A total of 1,378 disease targets were identified. By utilizing the microsignature platform to analyze intersections, 125 overlapping targets associated with potential active constituents related to RM diseases were identified (Figure 2A).

#### 2.2.3 Construction of PPI network

The analysis of the protein–protein interaction (PPI) network has revealed 10 principal targets: IL-6, PPARG, TP53, AKT1, ALB, BCL2, CASP3, EGFR, ESR1, and GAPDH. These targets exhibit a high degree of interconnectivity and are likely to be integral to the therapeutic effects of RM in the context of LI. Importantly, these targets are implicated in critical biological processes, including inflammation (IL-6, AKT1), apoptosis regulation (TP53, BCL2, CASP3), and metabolic modulation (PPARG, ESR1). This suggests that RM may provide multi-targeted protective effects in LI (Figure 2B).

#### 2.2.4 “Drug-component-target” interaction network and topological analysis

Network analysis has revealed six principal bioactive components in RM, namely adenosine, aloeemodin, emodin, kaempferol, paeonoside, and quercetin, which were identified due to their significant network connectivity (Figure 2C). These compounds demonstrated the most robust interactions with targets associated with liver injury, indicating their potential role as key active constituents responsible for mediating the therapeutic effects of RM.

#### 2.2.5 GO and KEGG pathway analysis

Following the importation of 125 intersection targets into the DAVID online database, a comprehensive screening yielded a total of 1,200 entries. Among these, 472 were categorized under biological processes, predominantly encompassing the negative regulation of apoptotic processes, intracellular receptor signaling pathways, positive regulation of gene expression, and negative regulation of gene expression, among others. Additionally, 62 entries were classified as cellular components, primarily involving receptor complexes, extracellular exosomes, cytosol, and mitochondria. Furthermore, 130 entries were identified under molecular functions, with a focus on protein binding, identical protein binding, zinc ion binding, and ATP binding, among other functions. The top 10 entries with the lowest *p*-values in each category were selected for visual analysis using MicroBioinformatics (Figure 2D). The Kyoto Encyclopedia of Genes and Genomes (KEGG) pathway enrichment analysis identified 103 pathways. After filtering out irrelevant pathways, such as those related to “cancer,” “lipids and atherosclerosis,” and “cytomegalovirus infection,” the top 20 pathways with the highest number of enriched targets were selected based on ascending *p*-values to create a bubble chart. The KEGG enrichment analysis indicated that *Rhododendron molle* is significantly associated with pathways pertinent to liver injury treatment, particularly involving signaling pathways such as the PI3K-Akt signaling pathway, VEGF signaling pathway, HIF-1 signaling pathway, and estrogen signaling pathway (Figure 2E).

#### 2.2.6 Molecular docking

**Figure 3:**
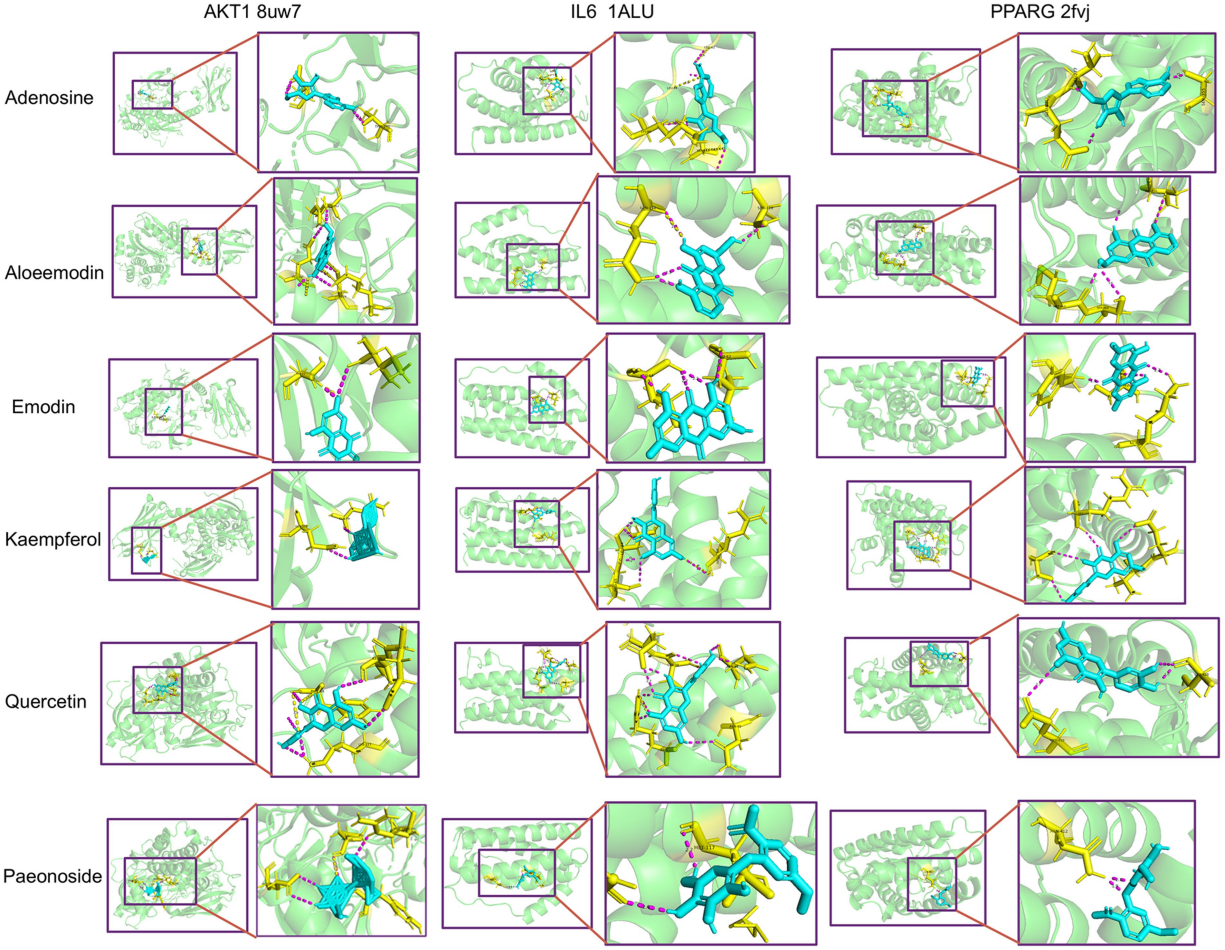
Molecular docking between key components and key targets.

In order to assess the binding affinity of the primary constituents of *Rhododendron molle* (Blume) G. Don with significant biological targets, we conducted molecular docking analyses. An analysis was conducted on the top 10 core targets identified by the “CytoHubba” plugin within the Cytoscape framework. From this analysis, six compounds—quercetin, kaempferol, emodin, aloe-emodin, adenosine, and paeonoside were selected for molecular docking studies with the key targets IL6, AKT1, and PPARG to assess their binding affinity and stability. The results of the molecular docking indicated a significant inverse relationship between the binding free energy and the stability of the ligand-receptor complexes. Specifically, conformations characterized by lower binding energies demonstrated enhanced thermodynamic stability. Comprehensive docking results are illustrated in Figure 3 and detailed in Table 2. The results show that the components aloeemodin and emodin have a high binding energy with the target IL-6, and aloeemodin also has a high binding energy with PPARG.

**Table 2:** Molecular docking results between the core components of RM and the core targets.

Gene name	PDB ID	Ligand	Binding energy (kcal/mol)
AKT1	8uw7	Adenosine	−3.08
IL6	1ALU		−4.8
PPARG	2fvj		−4.24
AKT1	8uw7	Aloeemodin	−6.11
IL6	1ALU		−6.56
PPARG	2fvj		−6.32
AKT1	8uw7	Emodin	−5.12
IL6	1ALU		−7.26
PPARG	2fvj		−7.3
AKT1	8uw7	Kaempferol	−4.62
IL6	1ALU		−5.17
PPARG	2fvj		−5.2
AKT1	8uw7	Paeonoside	−1.77
IL6	1ALU		−2.12
PPARG	2fvj		−1.92
AKT1	8uw7	Quercetin	−3.65
IL6	1ALU		−5.62
PPARG	2fvj		−4.67

### 2.3 The effects of RM on the pathological changes of liver tissue in rats with LI

**Figure 4:**
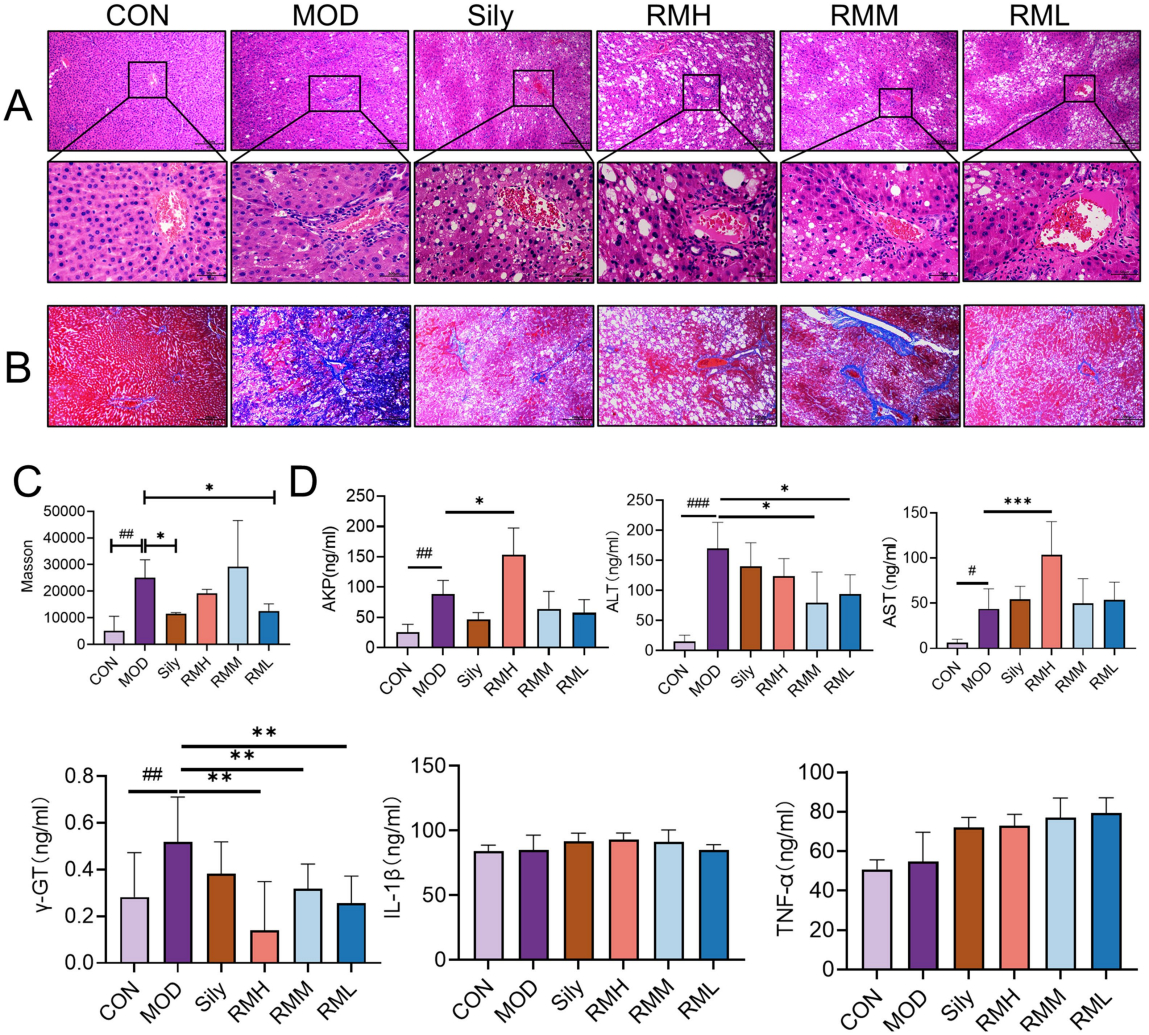
The effects of RM on the biochemical indexes of rats with LI. **(A)** HE staining of liver tissue under 100× and 400× microscopy. **(B)** Masson staining of liver tissue under 100× microscopy. **(C)** Scoring of Masson staining of liver tissue. **(D)** ELISA analysis of AKP, γ-GT, AST, ALT, TNF-α, and IL-1β in liver tissue. Compared with the MOD, ###*p* < 0.001, ##*p* < 0.01, and #*p* < 0.05; compared with the CON, ***p < 0.001, **p < 0.01, and *p < 0.05.

Histological examination of liver tissue using hematoxylin and eosin (HE) staining (Figure 4A) revealed distinct differences between the control group (CON) and the experimental groups. In the CON group, the liver lobules of the rats exhibited a uniform distribution, with liver cords arranged in a regular or striped configuration. Conversely, the model group (MOD) displayed significant lipid depletion within the liver cells, accompanied by areas of degeneration and necrosis, as well as extensive infiltration of granulocytes. In comparison to the MOD group, both the low-dose *Rhododendron molle* (Blume) G. Don group (RML) and the Silymarin group (Sily) demonstrated a reduction in inflammatory cell infiltration within the liver tissue. Additionally, there was a partial alleviation of lipid vacuolation in the liver cells, leading to a notable improvement in the extent of tissue damage. Notably, the pathological morphology of liver tissue in both the RML and Sily groups closely resembled that of the CON group.

Masson staining of liver tissue revealed (Figure 4B) that the CON group exhibited no significant fibrosis, while the MOD group displayed pronounced collagen fiber deposition within the liver tissue. In comparison to the MOD group, the liver tissue fibrosis in rats from the RML group, as well as those receiving medium-dose (RMM) and high-dose (RMH) *Rhododendron molle* (Blume) G. Don, and the Sily group, showed varying degrees of alleviation. The collagen deposition score in the MOD group was significantly elevated compared to the CON group (*p* < 0.05) (Figure 4C). Furthermore, the collagen deposition scores in the RML and Sily groups were both significantly lower than those in the MOD group (*p* < 0.05), with the RML group demonstrating a lesser degree of fibrosis and a collagen deposition score that approached that of the CON group.

### 2.4 The effects of RM on the biochemical indexes of liver tissue in rats with LI

To investigate the impact of RM on liver function and inflammatory markers in rats with LI, we performed enzyme-linked immunosorbent assays on liver tissue samples. The findings indicated (Figure 4D) that, in comparison to the CON group, the levels of alkaline phosphatase (AKP), gamma-glutamyl transpeptidase (γ-GT), aspartate aminotransferase (AST), and alanine aminotransferase (ALT) were significantly elevated in the MOD group. Conversely, the inflammatory markers tumor necrosis factor-alpha (TNF-α) and interleukin-1 beta (IL-1β) did not exhibit significant variations. Following the administration of three doses of RM to the rats with LI, the RMH demonstrated an increase in AKP levels compared to the MOD group, while no significant changes were observed in the RMM and RML groups. Additionally, the RMH group showed increased levels of AST and ALT in the liver tissue relative to the MOD group, and all three doses of RM resulted in a reduction of γ-GT levels in the liver tissue. However, the inflammatory markers TNF-α and IL-1β no significant changes across the LI and treatment groups. Based on the biochemical index results, it was observed that the RML group led to a reduction in ALT and γ-GT levels in rats with LI, and to a certain extent, also decreased AST and AKP levels. Consequently, we deduced that RML may have a more favorable effect on the repair of LI.

### 2.5 The effects of RM on the fecal metabolomics of rats with LI

#### 2.5.1 Analysis of the fecal metabolic profile of rats with LI by RM

**Figure 5:**
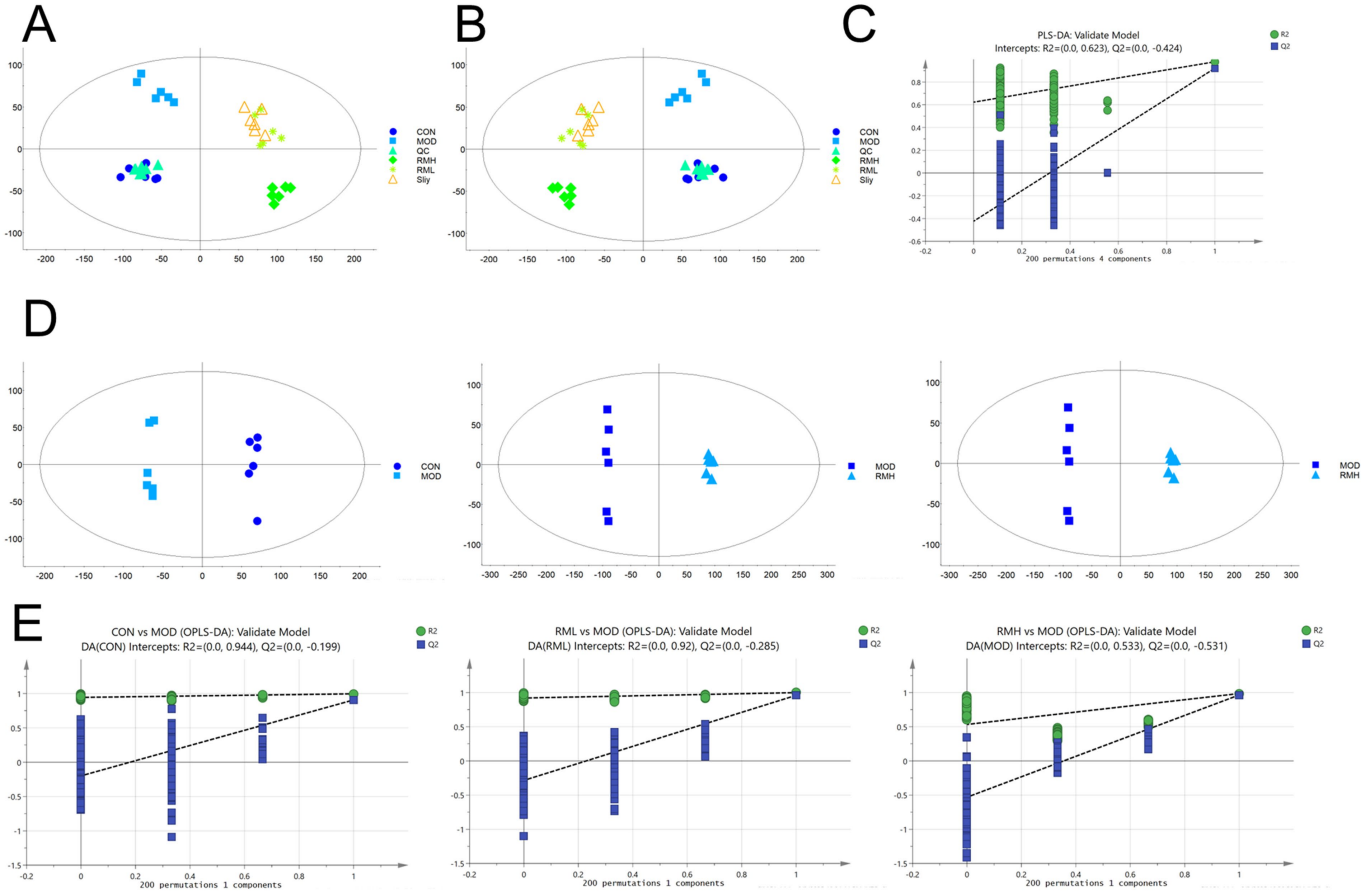
The metabolic profile analysis of RMH and RML. **(A)** PCA analysis of the each group under positive and negative ion modes. **(B)** PLSDA analysis of the each group under positive and negative ion modes. **(C)** PLS-DA test plots for each group. **(D)** OPLS-DA analysis of each group under positive and negative ion modes. **(E)** OPLS-DA tests for each group under positive and negative ion modes.

Based on the analysis of pathological sections and biochemical indices, we determined that the RML dosage was optimal. We hypothesized that the RMH dosage might exacerbate LI. Consequently, we undertook a non-targeted fecal metabolomics investigation comparing RMH and RML. The results of the principal component analysis (PCA) (Figure 5A) and partial least squares discrimination analysis (PLS-DA) (Figure 5B) indicated that metabolites within each group exhibited intra-group clustering and inter-group differentiation, the test results show (Figure 5C) that the *R*^2^ value is 0.623, indicating that the PLS-DA model has good predictive ability. To further evaluate the distinctions between the two groups, we employed orthogonal partial least squares discriminant analysis (OPLS-DA). The OPLS-DA model (Figure 5D) and the corresponding test results for each group (Figure 5E) revealed a notable distinction in metabolic products between the CON and MOD groups across both positive and negative ion modes. Furthermore, the *Q*^2^ values for the comparisons between the CON and MOD groups, as well as the MOD and RML groups, exceeded 0.9, suggesting that the predictive capacity of the OPLS-DA model is highly robust, thereby indicating notable differences in fecal metabolites between the two groups.

#### 2.5.2 Screening of differential metabolic components and cluster analysis

Utilizing a VIP >1 and a *p* < 0.05, differential metabolic components were identified among the CON and MOD groups, the MOD and RMH groups, as well as the MOD and RML groups. Specifically, a total of 84 differential metabolic components were detected in both positive and negative ion modes between the CON and MOD groups, among these, based on VIP >1 and a *p* < 0.05 while 55 differential metabolic components were identified in the MOD and RMH groups (Table 3), and 78 differential metabolic components were recognized in the MOD and RML groups (Table 4).

**Table 3:** Differential metabolic components were identified in the MOD and RMH group.

No.	Name	*m*/*z*	Formula	Retention time (min)	KEGG	VIP	Adducts	MS2	Trend	
									CON vs. MOD	RMH vs. MOD
1	24,25-Dihydroxyvitamin D	439.3205	C_27_H_44_O_3_	4.87	C01673	1.1166	M + Na	91.0542, 105.0699, 273.2213	↑###	↑***
2	24-Hydroxycholesterol	425.3415	C_27_H_46_O_2_	7.84	C13550	1.1228	M + Na	81.0699, 227.1794, 403.3517	↓###	↑***
3	2-Methylbutyrylglycine	160.0970	C_7_H_13_NO_3_	1.39	C03087	1.0264	M + H	114.0973, 160.0968	↑###	↓***
4	3alpha,7alpha-Dihydroxycoprostanic acid	457.3314	C_27_H_46_O_4_	4.87	C04554	1.0349	M + Na	69.0699, 71.0855	↑###	↑***
5	3-Hydroxyanthranilic acid	154.0501	C_7_H_7_NO_3_	2.76	C00632	1.2657	M + H	108.0449, 154.0504	↑###	↓***
6	4a-Carboxy-4b-methyl-5a-cholesta-8,24-dien-3b-ol	443.3522	C_29_H_46_O_3_	7.87	C15808	1.1324	M + H	55.0542, 81.0699, 443.3520	↓###	↑***
7	4alpha-Methylzymosterol	421.3463	C_28_H_46_O	6.06	C05103	1.2532	M + Na	55.0542, 81.0699, 341.3203	↑###	↓*
8	4-Trimethylammoniobutanoic acid	146.1177	C_7_H_15_NO_2_	1.46	C01181	1.1765	M + H	100.1121, 128.1070, 146.1176	↑###	↓***
9	5a-Cholestane-3a,7a,12a,25-tetrol	459.3473	C_27_H_48_O_4_	4.43	C05446	1.1859	M + Na	69.0669, 81.0699, 95.0855	↑###	↑***
10	7,8-Dihydropteroic acid	315.1230	C_14_H_14_N_6_O_3_	3.18	C00921	1.1531	M + H	123.0665, 133.0509, 149.0658	↓###	↑**
11	7-Dehydrocholesterol	407.3308	C_27_H_44_O	4.98	C01164	1.2515	M + Na	57.0699, 71.0855, 107.0855	↑###	↑**
12	7-Ketocholesterol	423.3256	C_27_H_44_O_2_	6.75	C05455	1.1155	M + Na	93.0699, 99.0855, 173.0961	↑###	↑*
13	9-Pentadecenoic acid	263.2006	C_15_H_28_O_2_	7.22	C00124	1.2356	M + Na	67.0542, 81.0699, 95.0855	↑###	↓***
14	Adrenic acid	355.2631	C_22_H_36_O_2_	4.32	C16527	1.2931	M + Na	81.0669, 93.0669, 273.3577	↓###	↑***
15	Arachidonic acid	327.2318	C_20_H_32_O_2_	3.85	C00219	1.0412	M + Na	121.0653, 161.0966, 179.1436	↓###	↑**
16	Campesterol	423.3621	C_28_H_48_O	9.32	C01789	1.2141	M + Na	55.0542, 81.0699, 105.0699	↑###	↓***
17	Cholesterol	409.3465	C_27_H_46_O	11.39	C00187	1.1152	M + Na	95.0855, 119.0855	↓###	↑**
18	Cortexolone	347.2218	C_21_H_30_O_4_	4.36	C05488	1.0226	M + H	91.0542, 175.1117, 259.2056	↑###	↑***
19	delta-Amorphene	205.1952	C_15_H_24_	9.32	C06394	1.2262	M + H	121.1012, 149.1325, 205.1951	↑###	↓***
20	Demethylphylloquinone	437.3393	C_30_H_44_O_2_	12.74	C13309	1.4392	M + H	55.0542, 105.0335, 1730591	↑###	↓***
21	Deoxycorticosterone	353.2075	C_21_H_30_O_3_	1.56	C03205	1.4305	M + Na	81.0699, 131.0855, 261.2213	↑###	↓***
22	Docosahexaenoic acid	329.2475	C_22_H_32_O_2_	4.51	C06429	1.1235	M + H	131.0855, 311.2369, 329.3475	↑###	↑**
23	Docosapentaenoic acid (22n-6)	353.2472	C_22_H_34_O_2_	4.00	C16513	1.0670	M + Na	79.0542, 81.0699, 157.1012	↓###	↑***
24	Farnesol	245.1900	C_15_H_26_O	9.32	C09621	1.2648	M + Na	93.0699, 95.0855, 149.1325	↑###	↓***
25	Gamma-Tocopherol	439.3570	C_28_H_48_O_2_	7.22	C02483	1.2963	M + Na	95.0497, 123.1174, 151.0759	↑###	↓***
26	Glutaminylhydroxyproline	260.1245	C_10_H_17_N_3_O_5_	2.45	C05939	1.0368	M + H	132.0655, 197.0921, 260.1241	↓###	↑***
27	Glycerophosphocholine	258.1098	C_8_H_20_NO_6_P	1.32	C00670	1.2000	M + H	89.0964, 184.0733	↓###	↓***
28	Hypogeic acid	255.2319	C_16_H_30_O_2_	7.22	C08362	1.3047	M + H	93.0699, 107.0855, 149.1325	↑###	↑*
29	Linoleic acid	281.2475	C_18_H_32_O_2_	9.17	C01595	1.1913	M + H	245.2264, 263.2369, 287.2745	↑###	↑**
30	Lipoamide	228.0505	C_8_H_15_NOS_2_	2.72	C05655	1.3862	M + Na	71.0861, 98.0970, 114.0919	↑###	↓***
31	Medroxyprogesterone	345.2424	C_22_H_32_O_3_	3.85	C00861	1.0861	M + H	259.2056, 271.2056, 327.2319	↓###	↑**
32	N6,N6,N6-Trimethyl-L-lysine	189.1599	C_9_H_20_N_2_O_2_	1.15	C03793	1.3251	M + H	268.1338, 286.1143	↑###	↓**
33	N-Acetylgalactosamine	222.0974	C_8_H_15_NO_6_	1.46	C01074	1.1158	M + H	186.0761, 204.0866, 222.0972	↓###	↓***
34	Palmitaldehyde	263.2370	C_16_H_32_O	9.64	C00517	1.1684	M + Na	81.0699, 95.0855, 97.1012	↑###	↓***
35	Prostaglandin E2	353.2304	C_20_H_32_O_5_	4.94	C00584	1.1030	M + H	115.1123, 335.2222, 353.2328	↓###	↑**
36	Retinal	285.2213	C_20_H_28_O	9.32	C00376	1.3076	M + H	67.0542, 81.0699, 91.0542	↑###	↓***
37	Secoisolariciresinol	363.1801	C_20_H_26_O_6_	3.74	C03958	1.1751	M + H	105.0355, 327.1591, 363.1802	↑###	↑**
38	Sphingosine	300.2896	C_18_H_37_NO_2_	5.49	C00319	1.1075	M + H	81.0335, 107.0491, 135.0441	↑###	↑**
39	Tetracosapentaenoic acid (24:5n-6)	359.2945	C_24_H_38_O_2_	8.06	C16533	1.2476	M + H	135.0855, 263.2006, 359.2945	↓###	↑***
40	Trigonelline	138.0552	C_7_H_7_NO_2_	3.08	C01004	1.1477	M + H	96.0813, 128.1075, 138.0555	↓###	↓***
41	13-L-Hydroperoxylinoleic acid	311.2227	C_18_H_32_O_4_	6.15	C04717	1.0814	M − H	71.0855, 95.0855, 135.1168	↓###	↑**
42	3-(3-Hydroxyphenyl)propanoic acid	165.0548	C_9_H_10_O_3_	3.54	C11457	1.1870	M − H	107.0491, 121.0648, 149.0597	↓###	↓***
43	1beta-Hydroxycholic acid	423.2754	C_24_H_40_O_6_	3.97	C01094	1.3768	M − H	83.0502, 423.2752	↓###	↑***
44	alpha-Linolenic acid	323.2226	C_18_H_30_O_2_	7.38	C06427	1.2566	M + FA − H	70.0287, 72.0444, 84.0808	↓###	↑***
45	Ascorbic acid	175.0239	C_6_H_8_O_6_	1.37	C01041	1.3705	M − H	59.0113, 99.0082, 131.0344	↓###	↓***
46	Deoxycytidine	272.0888	C_9_H_13_N_3_O_4_	2.32	C00881	1.4499	M + FA − H	79.0296, 95.0609, 126.0667	↑###	↓***
47	D-Xylose	195.0503	C_5_H_10_O_5_	1.33	C00181	1.1794	M + FA − H	105.0546, 151.0601	↑###	↓**
48	Glycerylphosphorylethanolamine	214.0481	C_5_H_14_NO_6_P	1.26	C01233	1.1697	M − H	96.9696, 122.0012	↓###	↓**
49	Homovanillic acid	181.0499	C_9_H_10_O_4_	3.02	C05582	1.0212	M − H	92.9977, 107.0497, 123.0082	↓###	↓**
50	Indolelactic acid	204.0660	C_11_H_11_NO_3_	3.58	C02043	1.0262	M − H	130.0655, 160.0757, 188.0706	↓###	↓*
51	Kynurenic acid	188.0345	C_10_H_7_NO_3_	3.05	C01717	1.4299	M − H	65.0392, 144.0449, 172.0399	↓###	↓***
52	N-Glycolylneuraminic acid	324.0936	C_11_H_19_NO_10_	1.33	C03410	1.2850	M − H	59.0138, 87.0088	↓###	↓***
53	Pantetheine	277.1232	C_11_H_22_N_2_O_4_S	3.02	C00831	1.4477	M − H	101.0720, 129.0492	↑###	↓***
54	Glycogen	665.2143	C_24_H_42_O_21_	1.54	C01613	1.2048	M − H	59.0138, 71.0139	↓###	↑**
55	Tyrosol	137.0597	C_8_H_10_O_2_	3.05	C06044	1.1283	M – H	65.0033, 91.0553, 93.0346	↓###	↓***

Compared with the MOD, ###*p* < 0.001, ##*p* < 0.01, and #*p* < 0.05; compared with the CON, ***p < 0.001, **p < 0.01, and *p < 0.05.

**Table 4:** Differential metabolic components were identified in the MOD and RML group.

No.	Name	*m*/*z*	Formula	Retention time (min)	KEGG	VIP	Adducts	MS2	Trend	
									CON vs. MOD	RML vs. MOD
1	Tetracosapentaenoic acid (24:5n-6)	359.2945	C_24_H_38_O_2_	8.06	C16533	1.3968	M + H	135.0855, 263.2006, 359.2945	↓###	↑***
2	Secoisolariciresinol	363.1801	C_20_H_26_O_6_	3.74	C03958	1.1692	M + H	105.0355, 327.1591, 363.1802	↑###	↑***
3	Medroxyprogesterone	345.2424	C_22_H_32_O_3_	3.85	C00861	1.3157	M + H	271.2056, 327.2340, 345.2433	↓###	↑***
4	Maslinic acid	473.3630	C_30_H_48_O_4_	4.94	C15802	1.0657	M + H	427.3571, 455.3520, 473.3625	↑###	↓**
5	Malonylcarnitine	248.1142	C_10_H_17_NO_6_	2.69	C01594	1.1613	M + H	248.1129	↓###	↑***
6	Lipoamide	228.0505	C_8_H_15_NOS_2_	2.72	C05655	1.3448	M + Na	71.0861, 98.0970, 114.0919	↑###	↓***
7	L-beta-aspartyl-L-leucine	247.1291	C_10_H_18_N_2_O_5_	2.48	C11512	1.4171	M + H	70.0287, 72.0444, 84.0808	↓###	↓***
8	Hypogeic acid	255.2319	C_16_H_30_O_2_	7.22	C08362	1.0344	M + H	93.0699, 107.0855, 149.1325	↑###	↑**
9	Glutaminylhydroxyproline	260.1245	C_10_H_17_N_3_O_5_	2.45	C05939	1.2102	M + H	132.0655, 197.0921, 260.1241	↓###	↑***
10	Farnesol	245.1900	C_15_H_26_O	11.39	C09621	1.0946	M + Na	93.0699, 95.0855, 149.1325	↓###	↑**
11	Docosapentaenoic acid (22n-6)	353.2472	C_22_H_34_O_2_	4.00	C16513	1.4390	M + Na	79.0542, 81.0699, 157.1012	↓###	↑***
12	delta-Amorphene	205.1952	C_15_H_24_	9.32	C06394	1.4598	M + H,	121.1012, 149.1325, 205.1951	↑###	↓***
13	Carisoprodol	261.1810	C_12_H_24_N_2_O_4_	1.29	C00341	1.1864	M + H	86.0600, 95.0855	↑###	↓***
14	9-Pentadecenoic acid	263.2006	C_15_H_28_O_2_	7.22	C00124	1.3813	M + Na	67.0542, 81.0699, 95.0855	↑###	↓***
15	8-iso-15-keto-PGE2	373.2006	C_20_H_30_O_5_	5.27	C00427	1.2810	M + Na	81.0699, 95.0491, 223.1329	↑###	↓***
16	7-Ketocholesterol	423.3256	C_27_H_44_O_2_	6.75	C05455	1.3380	M + Na	93.0699, 99.0855, 173.0961	↑###	↑***
17	5beta-Coprostanol	411.3620	C_27_H_48_O	10.50	C11455	1.1535	M + Na	95.0855, 147.1168, 161.1325	↓###	↑**
18	5a-Cholestane-3a,7a,12a,25-tetrol	459.3473	C_27_H_48_O_4_	4.43	C05446	1.3778	M + Na	69.0669, 81.0699, 95.0855	↑###	↑***
19	2-Methylbutyrylglycine	160.0970	C_7_H_13_NO_3_	1.39	C03087	1.3216	M + H	114.0973, 160.0968	↑###	↓***
20	24,25-Dihydroxyvitamin D	439.3211	C_27_H_44_O_3_	7.08	C01673	1.4080	M + Na	91.0542, 105.0699, 273.2213	↓###	↑***
21	1,3-Dimethyluracil	141.0660	C_6_H_8_N_2_O_2_	2.10	C05828	1.0139	M + H	56.0495, 113.0709, 141.0659	↑###	↓*
22	3a,6b,7a,12a-Tetrahydroxy-5b-cholanoic acid	423.2754	C_24_H_40_O_6_	3.97	C01094	1.3874	M − H	83.0502, 423.2752	↓###	↑***
23	4-ene-Valproic acid	187.0968	C_8_H_14_O_2_	3.61	C16648	1.0106	M + FA − H	97.1023, 141.0921	↓###	↓**
24	4-Hydroxybenzoic acid	183.0292	C_7_H_6_O_3_	2.73	C00156	1.3295	M + FA − H	97.1023, 141.0921	↑###	↓***
25	9,10-DHOME	313.2385	C_18_H_34_O_4_	8.03	C14828	1.1929	M − H	109.1023, 123.1025, 313.2384	↓###	↑**
26	alpha-Ketoisovaleric acid	161.0446	C_5_H_8_O_3_	1.47	C00141	1.3346	M + FA − H	71.0502, 115.0401	↓###	↓***
27	Ascorbic acid	175.0239	C_6_H_8_O_6_	1.37	C01041	1.3552	M − H	59.0113, 99.0082, 131.0344	↓###	↓***
28	Bilirubin	629.2602	C_33_H_36_N_4_O_6_	3.93	C00486	1.3730	M + FA − H	468.2493, 593.3334	↑###	↓***
29	Chenodeoxycholic acid	391.2853	C_24_H_40_O_4_	6.80	C02528	1.4530	M − H	343.2637, 391.2848	↑###	↑***
30	Deoxycytidine	272.0888	C_9_H_13_N_3_O_4_	2.32	C00881	1.4424	M + FA − H	79.0296, 95.0609, 126.0667	↑###	↓***
31	Glutaric acid	131.0339	C_5_H_8_O_4_	2.70	C00489	1.1688	M − H	53.0027, 87.0446	↓###	↓***
32	Homovanillic acid	181.0499	C_9_H_10_O_4_	3.02	C05582	1.0288	M − H	92.9977, 107.0497, 123.0082	↓###	↓***
33	Hydrocinnamic acid	195.0656	C_9_H_10_O_2_	3.54	C05629	1.3480	M − H	97.0290, 119.0133	↑###	↓***
34	Indole	162.0551	C_8_H_7_N	3.05	C00463	1.2874	M − H	116.05	↑###	↓***
35	Indoleacetic acid	220.0610	C_10_H_9_NO_2_	3.19	C00954	1.3338	M + FA − H	64.0187, 88.0187	↑###	↓***
36	N-Glycolylneuraminic acid	324.0936	C_11_H_19_NO_10_	1.33	C03410	1.3445	M − H	59.0138, 87.0088	↓###	↓***
37	Pantetheine	277.1232	C_11_H_22_N_2O_4_S_	3.02	C00831	1.4266	M − H	101.0720, 129.0492	↑###	↓***
38	Pregnanediol 3-O-glucuronide	541.3018	C_27_H_44_O_8_	3.44	C03033	1.3295	M + FA − H	59.0138, 71.0139, 467.2650	↓###	↑***
39	Saccharopine	321.1303	C_11_H_20_N_2_O_6_	1.33	C00449	1.2116	M + FA − H	102.0560, 128.0717, 130.0873	↑###	↓***
40	Tyrosol	137.0597	C_8_H_10_O_2_	3.05	C06044	1.1706	M − H	65.0033, 91.0553, 93.0346	↓###	↓***
41	Glycerophosphocholine	258.1098	C_9_H_9_NO6	1.32	C00670	1.2862	M + H	89.0964, 184.0733	↓###	↓***
42	Linoleic acid	279.2328	C_5_H_10_O_5_	8.54	C01595	1.1802	M − H	245.2264, 263.2369, 287.2745	↓###	↑***
43	Docosahexaenoic acid	329.2475	C_30_H_48_O_3_	4.51	C06429	1.2206	M + H	131.0855, 311.2369, 329.3475	↑###	↑***
44	Deoxycorticosterone	331.2230	C_30_H_44_O_2_	2.79	C03205	1.2485	M + H	81.0699, 131.0855, 261.2213	↑###	↓***
45	Trigonelline	138.0551	C_28_H_46_O	1.46	C01004	1.2692	M + H	96.0813, 128.1075, 138.0555	↑###	↓***
46	Cholesterol	409.3459	C_28_H_44_O_3_	8.24	C00187	1.2915	M + Na	95.0855, 119.0855	↓###	↑***
47	4a-Carboxy-4b-methyl-5a-cholesta-8,24-dien-3b-ol	487.3430	C_28_H_44_O	5.93	C15808	1.2578	M + FA − H	55.0542, 81.0699, 443.3520	↑###	↑***
48	Morphine	286.1440	C_27_H_46_O_4_	3.60	C01516	1.1682	M + H	268.1338, 286.1143	↑###	↓**
49	Niacinamide	123.0556	C_27_H_46_O_2_	2.10	C00153	1.1167	M + H	80.0500, 105.0453, 123.0558	↑###	↓**
50	Indolelactic acid	206.0815	C_24_H_42_O_21_	3.57	C02043	1.1269	M + H	130.0655, 160.0757, 188.0706	↓###	↓**
51	N-Acetylgalactosamine	222.0974	C_22_H_34_O_2_	1.46	C01074	1.3982	M + H	186.0761, 204.0866, 222.0972	↓###	↓***
52	Adrenic acid	355.2631	C_21_H_27_ClN_2_O_2_	4.32	C16527	1.1882	M + Na	81.0669, 93.0669, 273.3577	↓###	↑**
53	Palmitaldehyde	263.2370	C_21_H_24_O_7_	9.64	C00517	1.2755	M + Na	81.0699, 95.0855, 97.1012	↑###	↓***
54	Stachyose	665.2143	C_21_H_24_F_3_N_3_S	1.54	C01613	1.1931	M − H	59.0138, 71.0139	↓###	↓**
55	N6,N6,N6-Trimethyl-L-lysine	189.1599	C_20_H_30_O_5_	1.15	C03793	1.1939	M + H	268.1338, 286.1143	↑###	↓***
56	Sphingosine	300.2896	C_20_H_28_O	5.49	C00319	1.3041	M + H	81.0335, 107.0491, 135.0441	↑###	↑***
57	13-OxoODE	295.2268	C_20_H_22_O_6_	4.94	C14765	1.1514	M + H	93.0699, 135.0804, 277.2162	↓###	↑***
58	3alpha,7alpha-Dihydroxycoprostanic acid	457.3314	C_20_H_20_O_5_	4.87	C04554	1.3042	M + H	69.0699, 71.0855	↑###	↓***
59	24-Hydroxycholesterol	425.3414	C_20_H_20_O_5_	8.89	C13550	1.3081	M + H	81.0699, 227.1794, 403.3517	↓###	↑***
60	7-Dehydrocholesterol	407.3308	C_20_H_18_O_6_	4.98	C01164	1.3346	M + H	57.0699, 71.0855, 107.0855	↑###	↑***
61	1-Hexadecanol	265.2526	C_19_H_26_O_8_	8.85	C00823	1.2649	M + H	57.0699, 71.0855, 97.1012	↑###	↑***
62	Phenylacetic acid	137.0599	C_19_H_24_N_2_O_2_	3.57	C07086	1.4210	M + H	91.0542, 137.0597	↑###	↓***
63	Campesterol	423.3621	C_19_H_17_ClN_2_O	10.32	C01789	1.2543	M + Na	55.0542, 81.0699, 105.0699	↑###	↓***
64	gamma-Tocopherol	439.3572	C_18_H_32_O_16_	9.75	C02483	1.2077	M + H	95.0497, 123.1174, 151.0759	↑###	↓**
65	4alpha-Methylzymosterol	421.3463	C_18_H_32_O_16_	6.06	C05103	1.3037	M + H	55.0542, 81.0699, 341.3203	↑###	↓***
66	9,12,13-TriHOME	329.2333	C_18_H_26_O_5_	5.02	C14833	1.2074	M + H	57.0335, 75.0441, 183.1380	↓###	↑***
67	D-Xylose	195.0503	C_17_H_19_ClN_2_OS	1.33	C00181	1.1668	M + Na	105.0546, 151.0601	↓###	↓**
68	Prostaglandin E2	351.2176	C_16_H_30_O_2_	4.33	C00584	1.3459	M + Na	115.1123, 335.2222, 353.2328	↑###	↑***
69	Demethylphylloquinone	437.3393	C_16_H_17_N_3_O_4_S	12.74	C13309	1.3582	M + H,	55.0542, 105.0335, 1730591	↑###	↓***
70	Kynurenic acid	188.0345	C_16_H_12_O_5_	3.05	C01717	1.3599	M + H	65.0392, 144.0449, 172.0399	↓###	↓***
71	7,8-Dihydropteroic acid	313.1083	C_15_H_26_O	3.76	C00921	1.0705	M + Na	123.0665, 133.0509, 149.0658	↓###	↑**
72	alpha-Linolenic acid	323.2226	C_15_H_24_O	7.38	C06427	1.2550	M + Na	70.0287, 72.0444, 84.0808	↓###	↑***
73	Deoxyadenosine	274.0925	C_15_H_24_	2.45	C00559	1.4201	M + Na	94.0405, 136.0623	↓###	↓***
74	Retinal	285.2213	C_15_H_10_N_2_O_3_	9.32	C00376	1.4423	M + Na	67.0542, 81.0699, 91.0542	↑###	↓***
75	Cortexolone	347.2218	C_12_H_22_O_11_	4.36	C05488	1.2842	M + Na	91.0542, 175.1117, 259.2056	↑###	↑***
76	Arachidonic acid	327.2318	C_12_H_22_O_11_	3.85	C00219	1.3421	M + H	67.0548, 81.0704, 161.1330	↓###	↑***
77	3-Hydroxyanthranilic acid	154.0501	C_12_H_12_N_4_	2.76	C00632	1.2504	M + Na	108.0449, 154.0504	↑###	↓***
78	4-Trimethylammoniobutanoic acid	146.1177	C_10_H_12_O_4_	1.46	C01181	1.3439	M + H	100.1121, 128.1070, 146.1176	↑###	↓***

Compared with the MOD, ###*p* < 0.001, ##*p* < 0.01, and #*p* < 0.05; compared with the CON, ***p < 0.001, **p < 0.01, and *p < 0.05.

**Figure 6:**
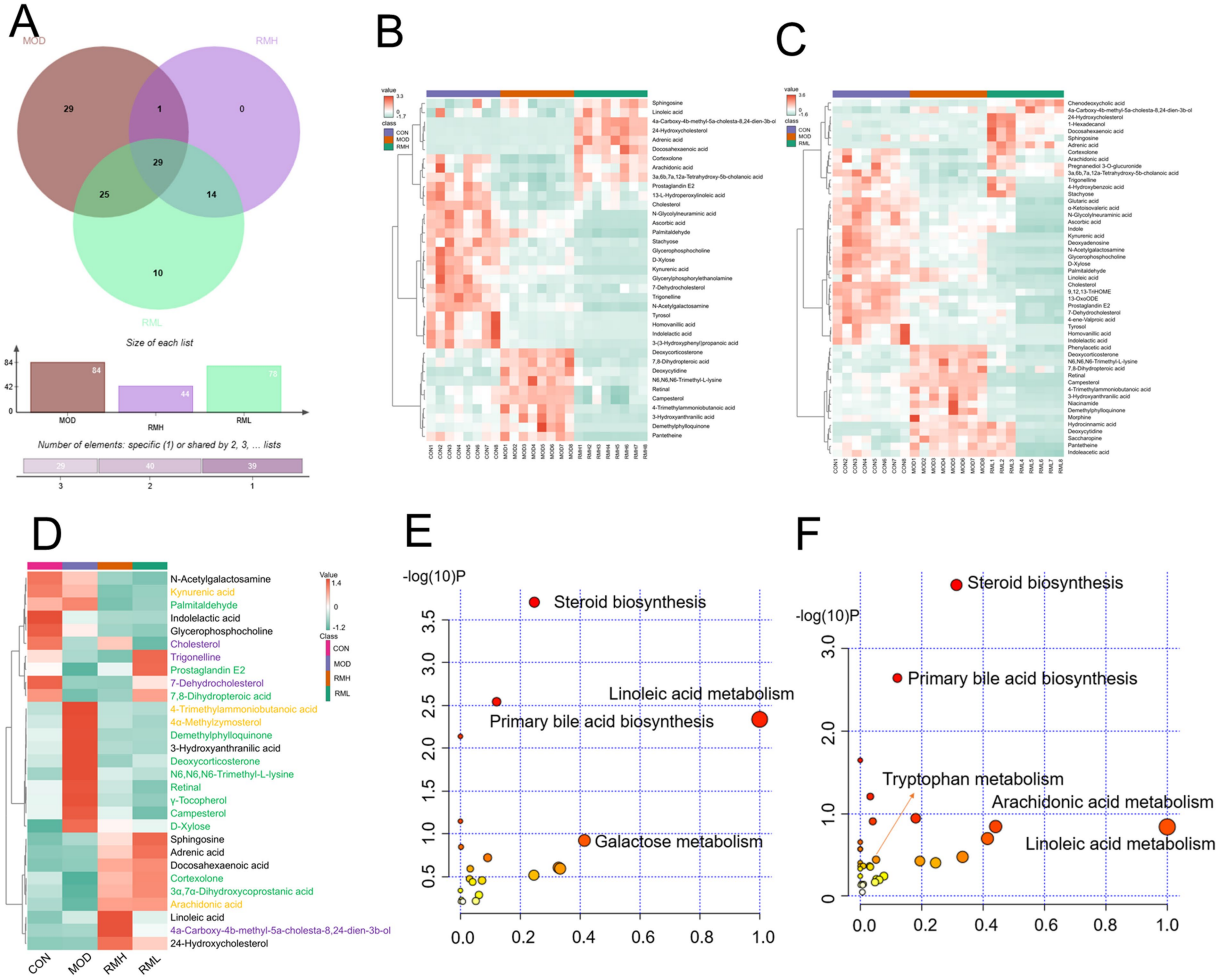
Analysis of fecal differential metabolic components. **(A)** OPLS-DA analysis of feces in positive and negative ion modes. **(B)** Venn analysis of differential metabolic components in each group. **(C)** Clustering heatmap of RMH of the CON and MOD. **(D)** Clustering heatmap analysis of RML group of the CON and MOD. **(E)** Clustering heatmap of common differential metabolic components of RMH and RML groups of the CON and MOD. **(F)** Metabolic pathways of the RMH group. **(G)** Metabolic pathways of the RML group. The color green signifies a notable upregulation or downregulation within the model group, which is subsequently reversed following treatment administration. The color purple denotes significant upregulation or downregulation in the model group, characterized by contrasting trends observed at both high and low dosage levels. Conversely, the color yellow represents significant upregulation or downregulation in the model group, exhibiting a consistent trend but varying degrees of change at high and low doses.

Cluster heatmap analysis was conducted based on the aforementioned differential metabolic components, The clustering analysis indicated that certain components of both the RML and RMH groups exhibited similarities to those of the CON group, with a greater number of components in the RML group demonstrating trends that aligned more closely with those of the CON group (Figures 6B,C). Furthermore, there were 34 common differential metabolic components identified across the four groups: CON, MOD, RMH, and RML (Figure 6A). A subsequent cluster analysis was performed on these 34 components (Figure 6D). RML and RMH exhibit distinct influences on the metabolic trends of various compounds, including 4a-Carboxy-4b-methyl-5a-cholesta-8,24-dien-3b-ol, 7-Dehydrocholesterol, cholesterol, and kynurenic acid. The observed variations in these metabolites may account for the differing effects associated with RMH and RML.

#### 2.5.3 Metabolic pathway analysis

To further investigate the effects of the selected metabolites and to identify the potential biochemical pathways impacted by LI, MetPA^1^ was utilized to elucidate the most pertinent pathways influenced by LI. As illustrated in Figures 6E,F, the metabolic pathways in the RMH group were predominantly enriched in steroid biosynthesis, linoleic acid metabolism, and arachidonic acid metabolism, among others. Conversely, the RML group was primarily associated with steroid biosynthesis, lysine degradation, pentose and glucuronate interconversions, linoleic acid metabolism, primary bile acid biosynthesis, and tryptophan metabolism, among other pathways. The variations in metabolites that exert reciprocal effects predominantly influence steroid biosynthesis, linoleic acid metabolism, and tryptophan metabolism, among other processes.

### 2.6 Integrated analysis of metabolomics and network pharmacology

**Figure 7:**
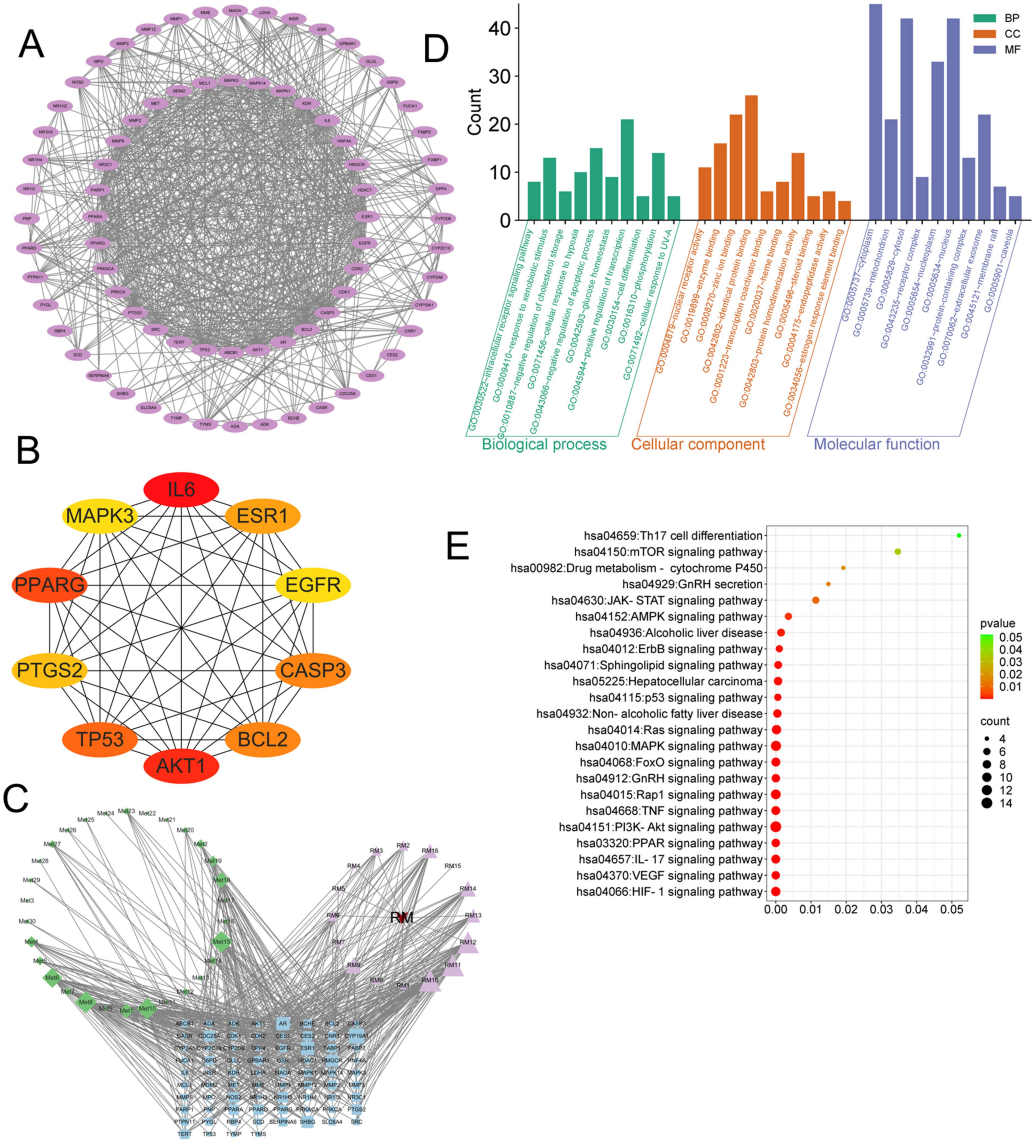
Integrated analysis of network pharmacology and metabolomics. **(A)** PPI network of RM components-disease-metabolite targets. **(B)** Top 10 targets after screening of core targets. **(C)** Network interaction of RM components-metabolites-disease. **(D)** GO enrichment analysis. **(E)** KEGG enrichment analysis.

We conducted an analysis of the intersection between 30 differential metabolites identified from the RML and their associated targets, alongside 125 drug-disease-metabolite targets predicted through network pharmacology (Figure 7A). This analysis yielded a total of 76 intersecting targets. The top 10 genes were identified using the Cytohubba plugin (Figure 7B), which included IL6, PPARG, AKT1, TP53, BCL2, CASP3, ESR1, PTGS2, MAPK3, and EGFR. Subsequently, we employed the “CytoNCA” plugin to identify the highest-ranked differential metabolic components (Figure 7C), which comprised 3α,7α-Dihydroxycoprostanic acid, arachidonic acid, cortexolone, deoxycorticosterone, pantetheine, and 13-OxoODE. The 76 core targets were then analyzed using the Metascape platform to perform Gene Ontology (GO) (Figure 7D) and KEGG (Figure 7E) analyses. The GO analysis encompassed three primary categories: biological process (BP), cellular component (CC), and molecular function (MF), which included a total of 209 BP, 42 CC, and 87 MF terms. The KEGG enrichment analysis indicated that RM positively influenced LI through 146 signaling pathways modulated by the differential metabolic components. Following the exclusion of pathways associated with cancer and those irrelevant to liver function, the most significant pathways identified were the PI3K-Akt signaling pathway, TNF signaling pathway, HIF-1 signaling pathway, IL-17 signaling pathway, PPAR signaling pathway, and mTOR signaling pathway, among others.

### 2.7 The impact of RML on the intestinal microbiota of rats with LI

#### 2.7.1 The impact of RML on the abundance of the intestinal microbiota of rats with LI

**Figure 8:**
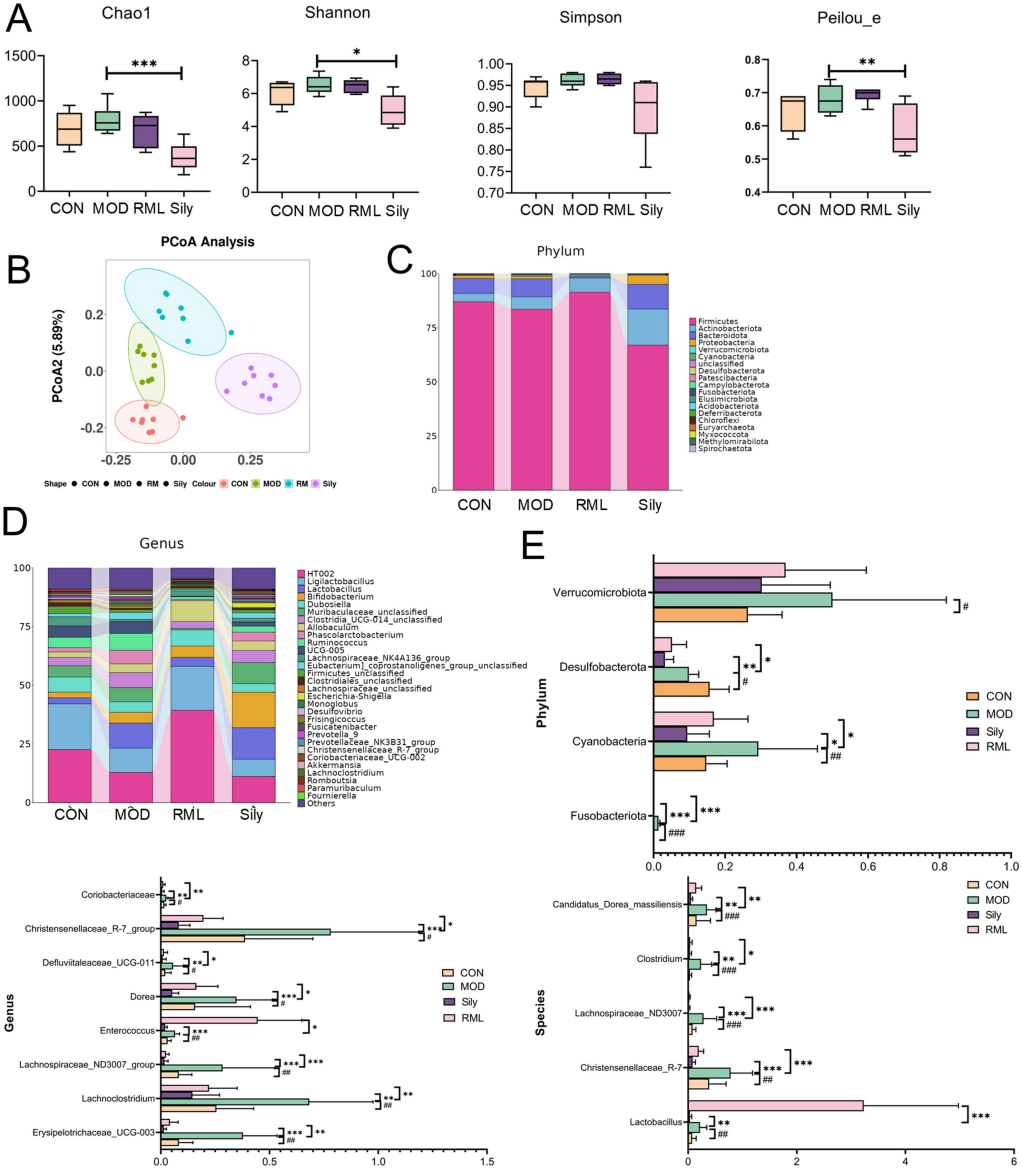
The impact of RML on the intestinal microbiota of rats with LI. **(A)** Alpha diversity analysis (Chao1 index, Simpson index, Shannon index, Peilou_e index). **(B)** Beta diversity analysis. **(C)** Stacked chart of differential microbiota at the phylum level. **(D)** Stacked chart of differential microbiota at the genus level. **(E)** Significance analysis of differential microbiota at the phylum, genus, and species levels. Compared with the MOD, ###*p* < 0.001, ##*p* < 0.01, and #*p* < 0.05; compared with the CON, ***p < 0.001, **p < 0.01, and *p < 0.05.

In light of the findings from pharmacodynamics and metabolomics, a comprehensive examination of the gut microbiota was performed for the group receiving the optimal RML. Alpha diversity analysis was utilized to assess the species diversity within individual samples, employing indices such as Chao1, Peilou_e, Shannon, and Simpson. Our findings (Figure 8A) indicate that RML did not significantly affect the richness and evenness of the intestinal microbiota in rats with LI. Following this, we performed a principal coordinate analysis (PCoA) on the operational taxonomic unit abundance matrices derived from the four experimental groups to evaluate the similarity among the samples and groups. The PCoA scores (Figure 8B) revealed a distinct separation of the MOD group from both the CON and RML groups, while showing a relative proximity to the Sily group.

#### 2.7.2 Analysis of intestinal microbial composition

The findings presented in the species annotation stacked charts (Figures 8C,D) indicate notable variations in the composition of microbial species across different taxa at both the phylum and genus levels.

We conducted an analysis of the microbial community changes at the phylum, genus, and species levels (Figure 8E). At the phylum level, a significant increase in the abundances of *Fusobacteriota*, *Cyanobacteria*, and *Desulfobacterota* was observed in comparison to the CON group. Following the administration of the Sily and RML groups, a marked reduction in the abundances of these phyla was noted. At the genus level, the administration of CCl_4_ resulted in a significant increase in the abundances of *Erysipelotrichaceae_UCG-003*, *Lachnoclostridium*, *Lachnospiraceae_ND3007_group*, *Enterococcus*, *Dorea*, *Defluviitaleaceae_UCG-011*, *Christensenellaceae_R-7_group*, and *Coriobacteriaceae*, among others. Conversely, both Sily and RML treatments led to a significant decrease in the abundances of these genera. To investigate the impact of RM on the intestinal microbiota at the species level, we conducted an analysis of the top 30 microbiota and performed a significance analysis on those that exhibited notable changes. The microbiota that demonstrated significant alterations are as follows: *Lactobacillus*, *Christensenellaceae_R-7*, *Lachnospiraceae_ND3007*, *Clostridium*, and *Candidatus_Dorea_massiliensis*. Subsequent to the administration of RM, there was a significant increase in the abundance of *Lactobacillus*, whereas the levels of *Christensenellaceae_R-7*, *Lachnospiraceae_ND3007*, *Clostridium*, and *Candidatus_Dorea_massiliensis* were restored to levels that approached normalcy.

**Figure 9:**
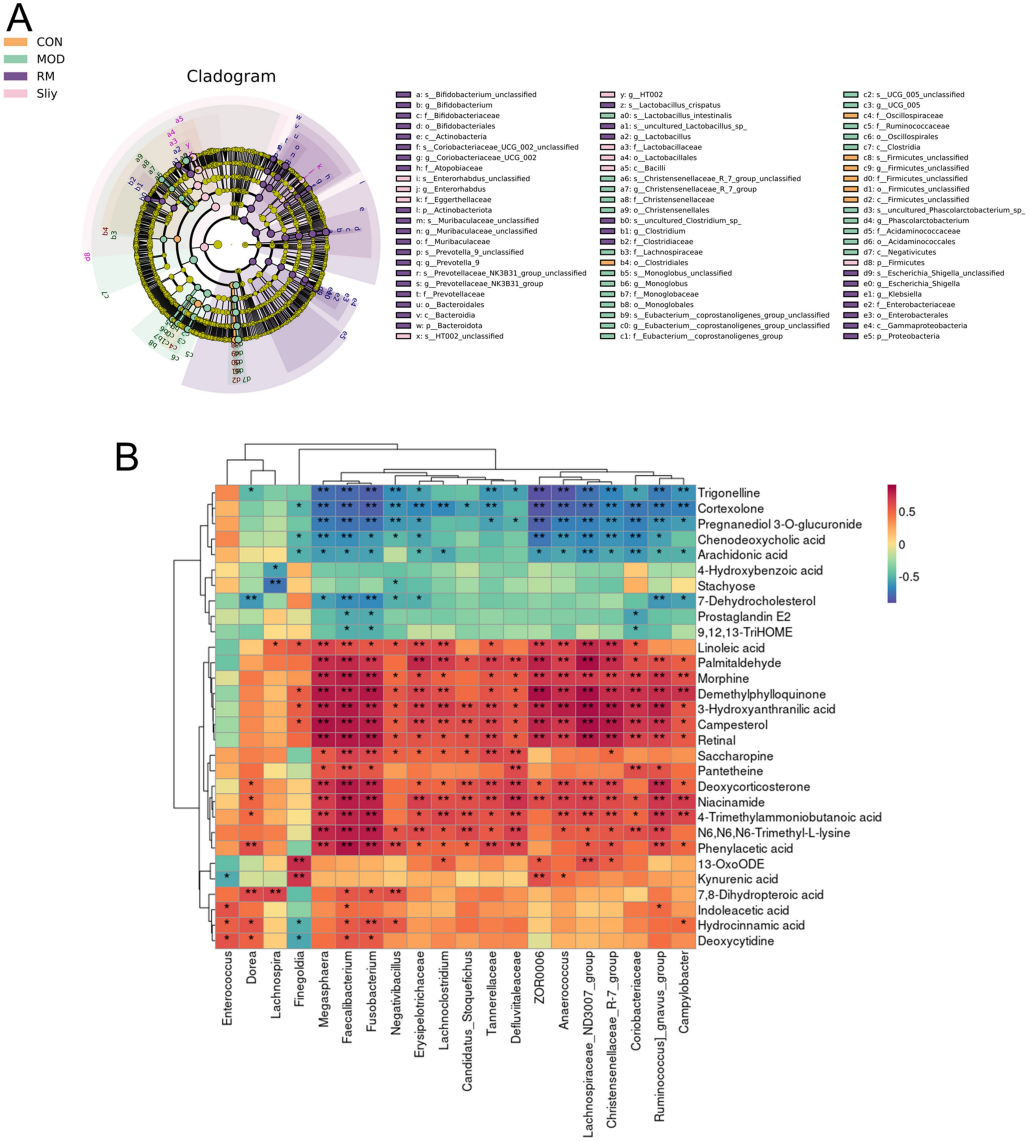
LEfSe analysis and correlation between differential metabolic components and gut microbiota. **(A)** LEfSe analysis of the intestinal microbiota. **(B)** Correlation analysis of the intestinal microbiota and the top 30 differential metabolic components in the RML group.

In this investigation, we employed the Linear discriminant analysis Effect Size (LEfSe) method to elucidate the significant variations in the composition of intestinal microbiota across different treatment groups, as illustrated in Figure 9A. By establishing a significance threshold of *p* < 0.05, we were able to identify a range of biomarkers that exhibited statistical significance. Within the CON group, we identified a total of seven microbiota types that were significantly enriched, including *Firmicutes*, *Oscillospiraceae*, and *Clostridiales*, which encompassed various taxonomic levels such as classes, orders, families, genera, and species within the Firmicutes phylum. The MOD group exhibited enrichment of 23 microbiota types, including *Clostridia*, *Ruminococcaceae*, *Negativicutes*, *Acidaminococcaceae*, *Phascolarctobacterium*, *UCG_005*, *Monoglobales*, and *Christensenellaceae*. In the RML group, 33 microbiota types were predominantly enriched, including *Bacteroidota*, *Lactobacillus*, *Actinobacteriota*, *Proteobacteria*, *Clostridium*, and *Atopobiaceae*. Lastly, the Sily group demonstrated enrichment of nine microbiota types, which included *Bacilli*, *Lactobacillaceae*, *HT002*, and *Enterorhabdus*.

### 2.8 Analysis of the correlation between differential metabolic components and gut microbiota

The association between intestinal microbiota and host metabolic processes was examined through the calculation of the Spearman correlation coefficient (Figure 9B). The analysis revealed that specific bacterial taxa exhibited significant correlations with a range of metabolites (*p* < 0.05). Notably, kynurenic acid demonstrated a positive correlation with *Finegoldia*, *ZOR0006*, and *Anaerococcus*. Conversely, 7-Dehydrocholesterol was found to have a negative correlation with *Erysipelotrichaceae*. Additionally, Deoxycytidine showed positive correlations with *Enterococcus*, *Dorea*, *Fusobacterium*, and *Faecalibacterium*. Furthermore, 7,8-Dihydropteroic acid was positively correlated with *Dorea*, *Lachnospira*, *Fusobacterium*, *Faecalibacterium*, and *Negativibacillus*. Lastly, palmitaldehyde exhibited positive correlations with a diverse array of bacterial groups, including *Fusobacterium*, *Faecalibacterium*, *Ruminococcus_gnavus_group*, *Anaerococcus*, *Tannerellaceae*, *Erysipelotrichaceae*, *Lachnoclostridium*, *Lachnospiraceae_ND3007_group*, *Campylobacter*, *Megasphaera*, *Defluviitaleaceae*, *Christensenellaceae_R-7_group*, *ZOR0006*, and *Coriobacteriaceae*.

## 3 Discussion

The liver, serving as the primary organ responsible for the metabolism of pharmaceuticals and toxic compounds within the human body, is particularly susceptible to injury from drugs, toxins, and viral infections (Su et al., 2023). In light of this context, the investigation of natural liver-protective compounds has emerged as a prominent area of research. This field is essential for the preservation of liver health and the effective regulation of various physiological processes, including metabolism, synthesis, and detoxification (Yang et al., 2019; Lu et al., 2016; Yang et al., 2015). RM is recognized as “Bashaga” within the context of Mongolian medicine and possesses a longstanding tradition of use in the treatment of liver ailments. Notably, the most pronounced hepatotoxic effects are observed following administration at dosages of 1.5 and 3 g/kg (Yao et al., 2011), consequently, we initiated a comprehensive investigation into the potential of RM to enhance LI, as well as the underlying mechanisms through which it exerts this effect. Our findings indicate that the RML, particularly at a dosage of 0.75 g/kg, exhibited the most pronounced efficacy in mitigating LI. Consequently, to assess the potential hepatotoxic effects of a higher dosage of RM, specifically 3 g/kg, we conducted subsequent fecal metabolomics analyses on both RMH and RML.

Through an analysis utilizing network pharmacology, the primary active constituents of RM that contribute to the amelioration of LI have been identified as adenosine, aloeemodin, emodin, kaempferol, paeonoside, and quercetin. The pivotal targets implicated in the therapeutic action of these components include AKT1, IL6, and PPARG. Prior research has demonstrated that in a model of acetaminophen-induced LI, kaempferol is capable of activating the Nrf2 signaling pathway, leading to the upregulation of Gpx4, inhibition of ferroptosis, and subsequent mitigation of LI. Furthermore, kaempferol may exert its effects through the modulation of targets such as AKT1. This study suggests that kaempferol may collaboratively contribute to the protective effects against LI by activating the Nrf2 signaling pathway and regulating targets like AKT1 (Mohamed et al., 2024).

At present, ALT, AST, AKP and γ-GT and are the primary biomarkers utilized for the clinical diagnosis of LI. In our investigation, the levels of these enzymes in the MOD group were significantly elevated in comparison to the CON group, thereby confirming the successful establishment of our experimental model. Additionally, the administration of RML resulted in a notable reduction in the levels of ALT and γ-GT in rats with LI, as well as a partial decrease in AKP levels. RM exhibits bidirectional regulation with a narrow therapeutic window. Low doses predominantly activate hepatic protective mechanisms, yielding significant efficacy. However, medium-to-high doses allow inherent hepatotoxic components to prevail, diminishing or reversing protection and reducing efficacy. At moderate doses, protective and toxic effects counterbalance, resulting in non-dose-dependent changes in classic liver enzymes.

Metabolomics serves as a significant methodology for the identification of novel biomarkers associated with LI (Gaggini et al., 2019). Based on the fecal metabolomics study of rats with LI, we screened the differential metabolic components of RMH and RML. Research has indicated that 13-OxoODE is directly linked to LI, this compound appears to facilitate the progression of LI by exacerbating oxidative stress, inducing endoplasmic reticulum stress, disrupting lipid metabolism, and promoting apoptosis (Zhang et al., 2017). In CECs, the synthesis of 13-Oxo-ODE can be stimulated through the metabolism of linoleic acid. Findings suggest 13-Oxo-ODE protects intestinal barrier integrity by suppressing inflammation. LPS-induced inflammation compromises barrier function, triggering dysbiosis, bacterial/metabolite translocation, immune activation, chronic inflammation, and potential systemic metabolic disturbances (Chopyk and Grakoui, 2020). The concentration of this metabolite was markedly reduced in the MOD group and exhibited a significant increase following the administration of RML, aligning with our findings.

Our investigation further demonstrated that RML markedly decreased bilirubin concentrations in LI rats. In the context of liver fibrosis, bilirubin metabolism frequently displays considerable irregularities, with increased serum bilirubin levels serving as a critical indicator of hepatic functional impairment. As liver fibrosis advances, the functionality of hepatocytes progressively declines, diminishing their capacity to uptake, conjugate, and eliminate bilirubin, which consequently leads to the accumulation of unconjugated bilirubin in the bloodstream (Surendran et al., 2020).

13-L-Hydroperoxylinoleic acid, an oxidative metabolite derived from linoleic acid, is integral to the process of hepatic fibrogenesis. In the context of nonalcoholic fatty liver disease and nonalcoholic steatohepatitis, increased concentrations of 13-L-Hydroperoxylinoleic acid contribute to the exacerbation of hepatocyte injury and the acceleration of fibrotic progression (Kasahara et al., 2023). The levels of 13-L-Hydroperoxylinoleic acid were markedly increased in the context of MOD, with a further significant elevation observed following the administration of RMH. Therefore, metabolomics research indicates that RML downregulated differential metabolic components such as 13-OxoODE, morphine, and niacinamide, while RMH showed the opposite trend.

This research incorporated the mechanisms of action associated with network pharmacology alongside a metabolomic analysis of RM in the context of treating LI. The findings indicated that the principal components of RM facilitated the amelioration of LI by modulating differential metabolites and engaging specific genes, including IL6, PPARG, and AKT1. These components exerted their therapeutic effects through various signaling pathways, notably the HIF-1 signaling pathway, IL-17 signaling pathway, PI3K-Akt signaling pathway, and TNF signaling pathway. Notably, during the process of LI amelioration, IL-6 emerged as a pivotal factor in the protection and repair of hepatic tissue, actively promoting the dedifferentiation and regenerative processes of hepatocytes (Li et al., 2023). The function of PPARG in enhancing LI is primarily demonstrated through its regulation of lipid metabolism, suppression of inflammatory responses, mitigation of oxidative stress, and facilitation of hepatocyte repair and regeneration (Wu et al., 2021). Furthermore, the function of AKT1 in enhancing LI is primarily evident through its facilitation of the upregulation of hepatocyte growth factor and vascular endothelial growth factor. This process results in the increased release of anti-inflammatory cytokines and growth factors within an inflammatory context, thereby contributing to a protective effect against immune-mediated hepatitis (Wang et al., 2024).

Through network pharmacology association of common differential metabolic components combined with RM components, we identified several critical pathways associated with the enhancement of LI. Significantly, the PI3K-Akt signaling pathway is implicated in the enhancement of LI. Activation of this pathway has been shown to suppress the synthesis of ECM and the activation of HSCs, while simultaneously promoting hepatocyte proliferation and inhibiting apoptosis, among other physiological functions (Jackson et al., 2008; Shamsan et al., 2024; Wang et al., 2022). Fatty acid metabolism is a critical component of liver function. It is integral to both the synthesis and storage of hepatic lipids, and it is also significantly associated with various pathological processes, including inflammatory responses, oxidative stress, and mitochondrial dysfunction (Kumar et al., 2023). The cytochrome P450 (CYP450) metabolic pathway of arachidonic acid (AA) is significant in the context of non-alcoholic fatty liver disease. Arachidonic acid influences the inflammatory response of hepatocytes via the phosphoinositide 3-kinase (PI3K)/Akt signaling pathway (Wang et al., 2025). Simultaneously, research has demonstrated that the activation of the PI3K-Akt signaling pathway exerts diverse influences on linoleic acid metabolism in liver tissue, encompassing antioxidant properties, regulation of lipid metabolism, responses to endoplasmic reticulum stress, and modulation of the FoxO signaling pathway (Li et al., 2024). Through the integration of metabolomics data with metabolic pathways and KEGG pathways, our findings suggest that RM has the potential to enhance lipid index by activating specific pathways and modulating fatty acid metabolic processes, including those related to linoleic acid and arachidonic acid metabolism.

The impairment of intestinal barrier function enhances the translocation of intestinal bacteria, which subsequently results in inflammation and the development of inflammatory conditions (Paulraj and Hojun, 2018). At the phylum level, the relative abundances of microbiota, including *Verrucomicrobiota*, *Desulfobacterota*, *Cyanobacteria*, and *Fusobacteriota*, were found to be elevated in rats subjected to CCl_4_-induced LI. Notably, *Fusobacteriota* is classified as a Gram-negative bacterium. In the intestinal microbial communities of healthy individuals, it typically constitutes a specific proportion; however, its abundance can fluctuate markedly under certain pathological conditions, such as colitis (Yu et al., 2022). In our investigation, the RML intervention effectively mitigated the dysbiosis of Fusobacteriota observed in rats with LI, restoring it to baseline levels. At the genus level, notable increases were observed in Erysipelotrichaceae_UCG-003, Lachnoclostridium, Lachnospiraceae_ND3007_group, Enterococcus, Dorea, Defluviitaleaceae_UCG-011, Christensenellaceae_R-7_group, and Coriobacteriaceae among the LI-affected rats, with varying degrees of reduction noted in the RML-treated group. Notably, Erysipelotrichaceae has been significantly elevated in various pathological conditions, including hyperlipidemia, chronic hepatitis B-related liver fibrosis, and non-alcoholic fatty liver disease (Matsushita et al., 2015; Neuschwander-Tetri, 2017). In our research, we observed that RML resulted in a reduction of *Erysipelotrichaceae* abundance in rats exhibiting LI. Furthermore, the variation in the relative abundance of *Lachnoclostridium* demonstrated a strong correlation with the severity of liver diseases. Specifically, in individuals diagnosed with alcoholic fatty liver disease (AFLD), the relative abundance of *Lachnoclostridium* at the genus level was significantly elevated in the AFLD cohort compared to the healthy control group (Chi et al., 2023). Moreover, *Lachnoclostridium* exhibited a notable increase in abundance within the cholestatic liver disease cohort and demonstrated a correlation with various serological markers indicative of liver pathology (Wu et al., 2020). At the species level, CCl_4_ administration resulted in a significant elevation of *Lactobacillus*, *Christensenellaceae_R-7*, *Lachnospiraceae_ND3007*, *Clostridium*, and *Candidatus_Dorea_massiliensis* in rat subjects. Notably, *Lactobacillus* exhibited a marked increase in comparison to the MOD group. *Lactobacillus* is intricately associated with liver pathologies, as it has the capacity to enhance the population of beneficial bacteria while suppressing the proliferation of pathogenic bacteria, thus contributing to the modulation of the intestinal microbiota balance (Kim et al., 2022). It has the potential to enhance the integrity of the intestinal barrier (Liu et al., 2022), additionally, it may mitigate liver inflammation by modulating the expression of inflammatory mediators (Yamazaki et al., 2022). The concentration of Lactobacillus exhibited a significant increase in the MOD group, suggesting that RML may contribute to the regulation of intestinal microbiota, enhancement of the intestinal barrier, and attenuation of inflammation levels in the large intestine.

The experimental findings indicated that the RML group exhibited significantly greater protective effects in alleviating LI compared to the RMH group. Biochemical assessments revealed that RML not only effectively diminished the area of collagen fiber deposition in the liver tissues of LI rats but also led to a substantial reduction in serum levels of ALT and γ-GT. Furthermore, metabolomic analysis elucidated that RML treatment resulted in a notable upregulation of 13-Oxo-ODE levels, alongside a downregulation of morphine and bilirubin expression. Importantly, the abnormal increase in 13-L-Hydroperoxylinoleic acid levels observed in the RMH group may correlate with the exacerbation of LI symptoms. Collectively, these results suggest that RML demonstrates superior efficacy in ameliorating LI. Nonetheless, the mechanisms by which potentially toxic components in RM influence LI, particularly regarding their role in exacerbating LI through specific pathways, necessitate further investigation in future studies. Initially, our research did not include validation of the RM reference standard. However, although 17 constituents were identified, their ADME properties lack pharmacokinetic validation. Importantly, predicted core targets (e.g., AKT1, IL-6, PPARG) require functional confirmation through cellular or genetic models. Finally, observed microbiota modulation necessitates causal verification via fecal transplantation to establish direct mechanistic links.

## 4 Materials and methods

*Rhododendron molle* (Blume) G. Don (RM) was purchased from Anguo Qi’ao Traditional Chinese Medicine Drinking Tablets Co. IL-1β, TNF-α, AST, ALT, AKP, γ-GT were purchased from Kote Biologicals, Mass spectinol, and acetonitrile were purchased from Thermo Fisher.

### 4.1 Drug extraction

Weigh 200 g of the RM. Subsequently, immerse it in 70% ethanol at a solid-to-liquid ratio of 1:10 for a duration of 30 min. Following this, perform reflux extraction under gentle boiling conditions for three cycles, each lasting 2 h. The resulting extract should then be subjected to suction filtration, followed by concentration and drying processes. The final extract is to be freeze-dried and subsequently ground into a powder for future applications.

### 4.2 Component analysis of RM

#### 4.2.1 Sample pretreatment

In the sample pretreatment procedure, 0.25 g of the powdered RM was accurately measured and subsequently combined with 10 milliliters of methanol. Following this, ultrasonic extraction was performed for a duration of 30 min. The resulting mixture was then subjected to centrifugation and filtration. Ultimately, the supernatant was collected for sample injection in subsequent analytical processes.

#### 4.2.2 Chromatographic conditions

The detection system was an Obitrap Exploris 120 (Thermo Fisher Scientific). The chromatographic column used was a Waters Acquity UPLC BEH with dimensions of 2.1100 mm and a particle size of 1.7 μm. The mobile phase consisted of two components: Mobile phase A was water containing 0.1% formic acid, and mobile phase B was acetonitrile. Gradient elution was adopted as follows: From 0 to 25 min, the proportion of mobile phase A changed from 99 to 1%, while that of mobile phase B changed from 1 to 99%. From 25 to 27 min, the proportion of mobile phase A was 1% and that of mobile phase B was 99%. From 27 to 28 min, the proportion of mobile phase A changed from 1 to 99%, and that of mobile phase B changed from 99 to 1%. From 28 to 30 min, the proportion of mobile phase A was 99% and that of mobile phase B was 1%. The injection volume was 2.0 μL, the flow rate was set at 0.3 mL/min, and the column temperature was maintained at 35.0°C.

#### 4.2.3 Mass spectrometry conditions

The ion source was a heated electrospray ionization (HESI). Both positive and negative ion detection modes were adopted. The sheath gas pressure was set at 35 Arb, the auxiliary gas volumetric flow rate was 10 Arb, the spray voltage was 3,500 V, the ion transfer tube temperature was 320°C, and the auxiliary gas temperature was 300°C. The scanning mode was Fullscan-ddMS2. The resolution of the full MS was 60,000, while the resolution of the dd-MS2 was 15,000. The scanning range was from 100 to 1,500, and the collision energy was 30%.

#### 4.2.4 Data processing

Progenesis QI was employed for baseline filtering, peak identification, integration, retention time correction, and peak alignment. The Xcalibur 4.0 software was utilized to analyze the data pertaining to the molecular ions and fragment ions associated with each component peak. By integrating the precise mass numbers of the compounds, the secondary fragmentation spectra, and referencing the HMDB database^2^ alongside the PubChem database,^3^ the primary chemical constituents of *Rhododendron molle* were identified.

### 4.3 Network pharmacology studies

#### 4.3.1 Screening of the targets of RM components

We performed a network-pharmacological analysis on the components of RM that were identified through MS/MS techniques. Following the retrieval of the Canonical SMILE sequences of these compounds from the PubChem database, we employed the SwissTargetPrediction database^4^ to forecast the potential targets associated with RM.

#### 4.3.2 Acquisition of LI-related disease targets and intersection target genes

Conduct a search for genes associated with liver injury (LI) in the GeneCards database^5^ using “Liver Injury” as the search term. Subsequently, compile a summary of the retrieved results to identify the complete set of genes related to LI. Employ Venny 2.1.0 (csic.es) to determine the intersection of genes between the target genes of the active components of RM and the genes associated with LI, and create a Venn diagram to visually represent these findings.

#### 4.3.3 Construction of the “drug-active component-potential target” network

In the Cytoscape 3.10.1 software, a network was developed to represent the interactions between drug-active components and their potential targets, which was then subjected to analysis. The significance of each node in relation to others, as well as the extent of each node’s influence, was evaluated using the Degree value. As a result, the primary components involved in the treatment of LI by RM were identified through a screening process that employed Degree values.

#### 4.3.4 Construction of the protein–protein interaction network

The genes that overlap between RM and LI were uploaded to the STRING database,^6^ with the organism specified as “*Homo sapiens*” This process generated a PPI network diagram, which was subsequently saved in “tsv” file format. The resulting data were then imported into Cytoscape version 3.10.1, where the top 10 core targets were identified utilizing the “Cytohubba” plugin for network analysis.

#### 4.3.5 Enrichment analysis of GO biological processes and KEGG pathways

Enrichment analyses for GO biological processes and KEGG pathways were performed using the DAVID database.^7^ Upon entering the database, users are prompted to select the “Functional Annotation” option. The predicted critical targets were subsequently uploaded to the DAVID database to facilitate the KEGG enrichment analysis. Users must designate “GENE SYMBOL” and specify “Human” to ensure the selection of “*Homo sapiens*” prior to data submission. Following this, the “KEGG Pathways” option is selected to download the relevant pathway files. The screening criteria established a significance threshold of *p* < 0.05, simultaneously, it is essential to eliminate extraneous pathways, including but not limited to “cancer,” “atherosclerosis,” and “proteoglycan signaling pathway in cancer,” with results organized by *p*-value to identify the top 20 pathways. The enrichment results were ultimately visualized using the online platform Bioinformatics,^8^ which offers tools for the graphical representation of the data.

#### 4.3.6 Molecular docking validation

Utilizing the Degree values from the network analysis, core components and high-ranking targets were identified for molecular docking studies, employing software tools such as AutoDock Tools and PyMOL. The two-dimensional structures of the small molecules were sourced from the PubChem database,^9^ and the Open Babel GUI software was employed to convert these structures into mol2 file format for subsequent applications. Following this, the three-dimensional structural files of the core targets were retrieved from the Protein Data Bank (PDB).^10^ Preliminary procedures, including the extraction of small molecules and the removal of hydrogen atoms, were conducted using PyMOL, with the resultant files saved for future reference. The small molecule and target protein data were then imported into AutoDockTools for the molecular docking process. Finally, PyMOL was utilized once more for the visual analysis of the docking outcomes.

### 4.4 Animal experimentation

#### 4.4.1 Materials

Silymarin purchased from Rigalon; *Rhododendron molle* (Blume)w G. Don was purchased from Anguo Qi’ao Traditional Chinese Medicine Drinking Tablets Co.; AKP, γ-GT, AST, ALT, TNF-α, IL-1β were purchased from Kurt Bio; Methanol and acetonitrile were purchased from Thermo Fisher.

#### 4.4.2 Animal experimentation and grouping

Sixty specific pathogen-free (SPF) male SD rats with a body weight of 180 ± 20 g (provided by (Beijing) Biotechnology Co., Ltd.) were acclimated for 7 days and then randomly divided into six groups: CON with 10 rats, MOD with 10 rats, RMH, RMM, RML each with 10 rats, and a Sily group with 10 rats. The license number for the experimental animals is: SCXK (Jing) 2019–0010. The housing conditions were maintained at a temperature of 22–26°C, approximately 33% humidity, and a 12-h light/dark cycle. The animal experiments were approved by the Medical Ethics Committee of Baotou Medical College, Inner Mongolia University of Science and Technology (approval number: Baotou Medical College Lunjian Animal 2021 No. 018). The experiment lasted for 14 days. The MOD group, RM group, and Sily group were subjected to modeling twice weekly (40% CCl_4_ + corn oil) on Mondays and Thursdays each week. The initial injection dose was 5 mg/kg, followed by subsequent injections of 3 mg/kg. The CON and MOD groups were given 0.1% sodium carboxymethyl cellulose solution by gavage daily; the Sily group was dissolved in 0.1% sodium carboxymethyl cellulose solution at a dosage of 50 mg/kg. According to the “Chinese Pharmacopoeia,” the recommended human oral dose of RM is 0.6–1.5 g. In this study, 1.5 g was designated as the medium dose, while doses 2-fold lower and 2-fold higher than the medium dose were defined as the low and high doses, respectively. Based on a standard human body weight of 60 kg, the equivalent doses for rats were calculated as follows: low dose = (0.75/60) × 6.3, medium dose = (1.5/60) × 6.3, and high dose = (3/60) × 6.3, which translates to crude drug dosages of 0.07875 g/kg, 0.0575 g/kg, and 0.315 g/kg, respectively. These were dissolved in 0.1% sodium carboxymethyl cellulose and administered by gavage.

#### 4.4.3 Treatment and sample collection

On the fourteenth day of the experimental protocol, following a period of fasting and water deprivation, fresh fecal samples were collected from the rats utilizing the extrusion method. A total of three samples were obtained and subsequently stored at −80°C in a freezer. Anesthesia was administered to the rats via intraperitoneal injection of 3% pentobarbital sodium at a dosage of 30 mg/kg. Following anesthesia, blood samples were extracted from the abdominal aorta of each group of rats. These blood samples were then subjected to centrifugation at 4°C at a speed of 3,500 r/min for a duration of 15 min to facilitate the separation of serum. The resulting supernatant serum was transferred into 250 μL centrifuge tubes for storage. Subsequently, the rats were euthanized through cervical dislocation, and the liver tissues were excised, rinsed with normal saline to eliminate residual blood, and sectioned into multiple portions, which were wrapped in aluminum foil. A segment of the liver tissue, approximately 2 cm from the hepatic portal region, was preserved in paraformaldehyde for future histological analysis, including HE staining and Masson’s trichrome staining.

### 4.5 Observation of hepatic tissue pathological changes using HE and Masson’s staining

After the liver tissues from each group of rats were fixed for a duration of 48 h, they underwent processing in an automated dehydration apparatus for gradient dehydration, followed by embedding. Subsequent to sectioning and dewaxing, the tissue sections were stained utilizing the HE and Masson’s staining kits in accordance with the manufacturer’s protocols. The HE staining technique was employed to examine the pathological morphology of the liver lobules, while Masson’s staining was utilized to assess the deposition of liver fibrosis and the distribution of fibrotic tissue. The percentage of collagen fiber area was quantified using Image-Pro Plus 6.0 software.

### 4.6 ELISA for the detection of liver function and inflammatory markers

Liver tissues were obtained from a − 80°C freezer and subsequently thawed on ice. A weight ratio of 1:9 was established, comprising liver tissue and physiological saline, and the tissue was homogenized while maintained on ice. Post-homogenization, the resultant mixture underwent centrifugation at 3,000 rpm for a duration of 10 min utilizing a centrifuge. The supernatant was then meticulously collected and processed in accordance with the protocols outlined in the ELISA kits designed for the assessment of liver function and inflammatory markers.

### 4.7 Fecal untargeted metabolomics analysis

#### 4.7.1 Sample preparation

Fecal samples were obtained from a −80°C freezer. Approximately 100 mg of fecal matter was transferred into a 1.5 mL centrifuge tube, to which 1,000 μL of 75% methanol was added. The resulting mixture was homogenized and agitated for 30 s, followed by sonication for a duration of 10 min. Subsequently, the mixture was centrifuged at 13,000 rpm, and the supernatant was filtered through a membrane to prepare the sample for fecal analysis. An injection volume of 4 μL was utilized for the analytical procedure.

#### 4.7.2 QC sample preparation

Carefully measure 100 mg of fecal samples from each group and combine them thoroughly to produce a homogenized composite sample.

#### 4.7.3 Chromatographic conditions

Chromatographic column: ACQUITY UPLCTM C18 column (100 mm × 2.1 mm i.d., 1.7 μm) (Waters Corporation, United States); Mobile phase: Mobile phase A is 0.1% acetonitrile, and mobile phase B is 0.1% formic acid in water; Column temperature: 40°C; Flow rate: 0.4 mL/min; Injection volume: 4 μL; Gradient elution method: 0–2 min, 99–50% A, 1–50% B; 3–10 min, 50% A to 1% A, 50% B to 99% B; 11–15 min, 1–99% A, 99% B to 1% B.

#### 4.7.4 Mass spectrometry conditions

Ion source: Electrospray ionization (ESI) source; Switching between positive and negative ion modes; Operating mode: Full scan-data dependent secondary scanning (Full MS-ddMS2); Mass spectrometry parameters are as follows: Sheath gas flow rate: 30 arb (ESI^+^), 35 arb (ESI^−^); Auxiliary gas flow rate: 10 arb; Spray voltage: 3.5 kV (ESI^+^), 3.0 kV (ESI^−^); Column temperature set to 35°C.

#### 4.7.5 Data processing

The raw data were analyzed using Progenesis QI software to obtain the normalized label-free results of metabolic peaks for each sample. The samples were normalized based on the total ion intensity of each chromatogram. The data matrix consisting of sample codes, RT-*m*/*z* pairs, and peak areas was imported into MassLynx V4.1 software for PCA, PLS-DA, and OPLS-DA. Based on the OPLS-DA analysis results of the control and model groups, a VIP list file was created to identify metabolic variables with VIP >1 and *p* < 0.05 from independent sample t-tests for further analysis. By combining public databases such as HMDB (see text footnote 2), KEGG,^11^ mbrole2,^12^ and relevant literature, the structures of potential biomarkers for LI were identified. PCA, PLS-DA, and OPLS-DA analyses were conducted using EZinfo 3.0 for Waters, and the validation analysis was performed using SIMCA 14.1. The names of the identified biomarkers were imported into the Metaboanalyst 6.0 website^13^ to analyze and determine the disrupted metabolic pathways. Metabolic pathways closely related to liver injury were obtained based on the criterion of impact >0.

### 4.8 Component-gene-disease-metabolite network

#### 4.8.1 Network construction

The differential metabolites identified between RM and LI are regarded as potential targets for RM in its interaction with LI. Following this, the overlapping targets are incorporated into Cytoscape version 3.10.3 to construct a “component-target-metabolite” interaction network.

#### 4.8.2 PPI analysis and core target screening

An interaction analysis of potential targets was performed utilizing the STRING database to examine proteins at a systemic level. We identified targets associated with RM components, LI components, and metabolic components, subsequently importing the intersecting targets into Cytoscape version 3.10.3. Employing the “Cytohubba” plugin, we identified the top 10 core targets and conducted a visual analysis of these targets.

#### 4.8.3 GO and KEGG enrichment analysis

The overlapping targets were uploaded to the Metascape database^14^ for GO enrichment and KEGG pathway analysis, in order to identify representative biological processes and pathways.

### 4.9 Fecal 16S rDNA analysis

#### 4.9.1 Sample preparation

In accordance with the protocols for 16S rDNA gene sequencing, Hangzhou Lianchuan Biotechnology Co., Ltd. employed the E.Z.N.A.^®^ Stool DNA Kit to extract DNA from rat intestinal content samples. Subsequent to the extraction process, agarose gel electrophoresis was utilized to assess the quality of the extracted DNA, while a UV spectrophotometer was employed for quantification purposes, thereby ensuring the accuracy and reliability of the obtained results.

#### 4.9.2 High-throughput 16S rDNA sequencing

The V3-V4 region of the prokaryotic small subunit (16S) rRNA gene was amplified using primers 341F (5′-CCTACGGGNGGCWGCAG-3′) and 805R (5′-GACTACHVGGGTATCTAATCC-3′). The total volume of the PCR amplification reaction mixture was 25 μL, which contained 25 ng of template DNA, 12.5 μL of a specific reagent, 2.5 μL of each primer, and ddH₂O (deionized distilled water) to adjust the volume. Subsequently, the PCR products were separated by 2% agarose gel electrophoresis to confirm the obtained DNA fragments. Throughout the DNA extraction process, ultrapure water was utilized instead of the sample solution. This was done to eliminate the possibility of false-positive PCR results and served as a negative control. The PCR products were purified by AMPure XT beads (Beckman Coulter Genomics, Danvers, MA, United States) and quantified using Qubit (Invitrogen, United States). The amplicon pool was then prepared for sequencing. The size and quantity of the amplicon library were, respectively, evaluated on an Agilent 2100 Bioanalyzer (Agilent, United States) and with a library quantification kit from Illumina (Kapa Biosciences, Woburn, MA, United States). Sequencing of the library was carried out on the NovaSeq PE250 platform.

#### 4.9.3 Data processing

The gut microbiota data were processed utilizing the QIIME2 software. Alpha diversity analysis was performed using Graphpad version 9.5. Beta diversity and species composition analyses were conducted on the Lianchuan Bioinformatics Cloud Platform.^15^ Additionally, the LEfSe analysis was executed on the Biosciences Cloud Platform,^16^ with the linear discriminant analysis (LDA) score established at 3.5.

### 4.10 Statistical methods

Data analysis was conducted using the SPSS 22.0 statistical software. Measurement data were expressed as the mean ± standard deviation ( $\bar{x}\pm s$ ). For comparisons among multiple groups, one-way analysis of variance (one-way ANOVA) was employed. When pairwise comparisons were needed, the *t*-test was utilized. A *p*-value less than 0.05 was considered to indicate a statistically significant difference.

## 5 Conclusion

This investigation systematically examined the mechanisms by which RM addresses LI through the integration of metabolomics, network pharmacology, and 16S rRNA methodologies. The findings indicated that RM, particularly the RML variant, demonstrated a protective effect against CCl_4_-induced LI by modulating various metabolic pathways, including Arachidonic acid metabolism, Linoleic acid metabolism, and Steroid biosynthesis. These results underscore the multi-target and multi-pathway nature of RM’s action in mitigating CCl_4_-induced LI. In contrast, RMH did not demonstrate any advantageous effects on LI as evidenced by biochemical markers and histopathological examinations. These findings suggest that RM may represent a promising therapeutic candidate for the treatment of CCl_4_-induced LI, with the identified targets potentially serving as viable candidates for future diagnostic or therapeutic interventions. According to our findings, the suggested clinical dosage is 0.07875 g/kg. Furthermore, the insights gained from the network pharmacology analysis of RM will inform the objectives of subsequent research endeavors.

## Data Availability

The original contributions presented in the study are publicly available. All raw Illumina sequencing data have been deposited in the NCBI SRA under BioProject accession number PRJNA1294298. Mass spectrometry data for the metabolomics metabolites are available in the article and Supplementary material.

## Footnotes

^1^ https://www.metaboanalyst.ca/MetaboAnalyst/home.xhtml

^2^ https://hmdb.ca/

^3^ https://pubchem.ncbi.nlm.nih.gov/

^4^ http://www.swisstargetprediction.ch

^5^ https://genealacart.genecards.org/

^6^ https://stringdb.org/

^7^ https://david.ncifcrf.gov/

^8^ https://www.bioinformatics.com.cn/login/

^9^ https://pubchem.ncbi.nlm.nih.gov/

^10^ https://www.rcsb.org/

^11^ http://www.genome.jp/kegg/

^12^ http://csbg.cnb.csic.es/mbrole2/index.php

^13^ http://www.metaboanalyst.ca/

^14^ www.metascape.org

^15^ https://www.omicstudio.cn/tool

^16^ https://www.bioincloud.tech/task-meta
