# Supplementary material for: Integrative network pharmacology, metabolomics and gut flora studies reveal mechanisms of action of Rhododendron molle (Blume) G. Don to ameliorate liver injury
Source: Front Microbiol. 2025 Sep 1;16:1570229. doi: 10.3389/fmicb.2025.1570229 (PMC12434124; doi:10.3389/fmicb.2025.1570229)
Supplement: Supplementary file 1 [file Supplementary_file_1.docx]

1. Citric Acid

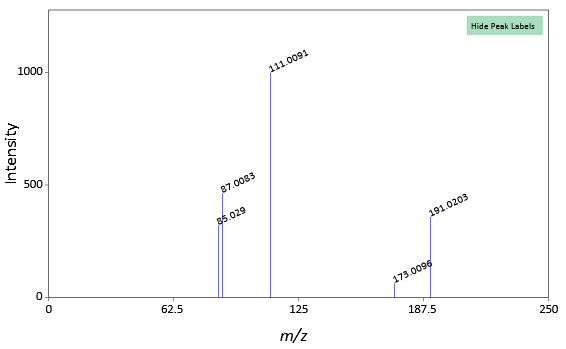


MS2：

111.0091、173.0096、191.0203

2. Heriguard

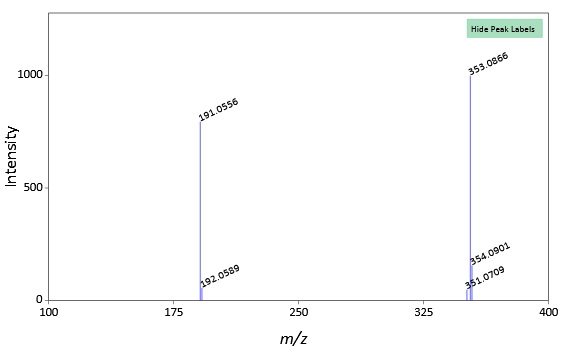


MS2：

191.0556、353.0866、351.0709、354.0901

3. 3-O-p-coumaroylquinic acid

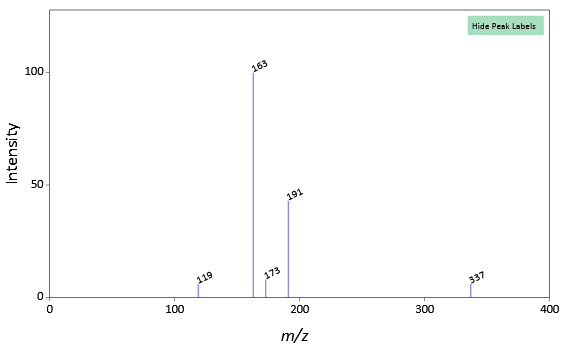


MS2：

163、337

4. Aloeemodin-Omega-O-Beta-D-Glucopyranoside


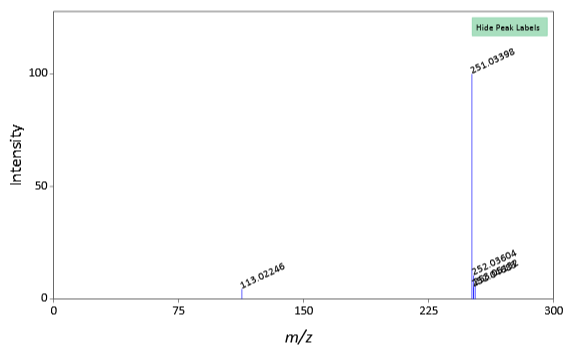


MS2：

251.03398、252.03604、253.05132

5. Oleanic acid

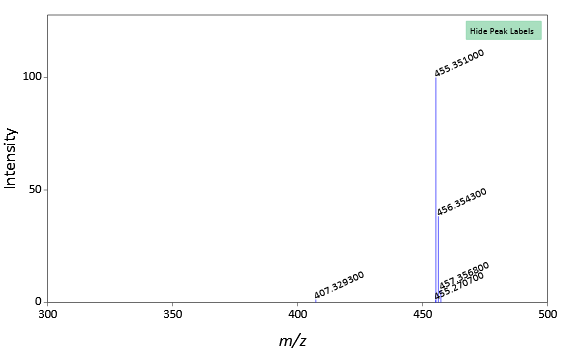


MS2：

455.270700、456.354300

6. Kaempferol


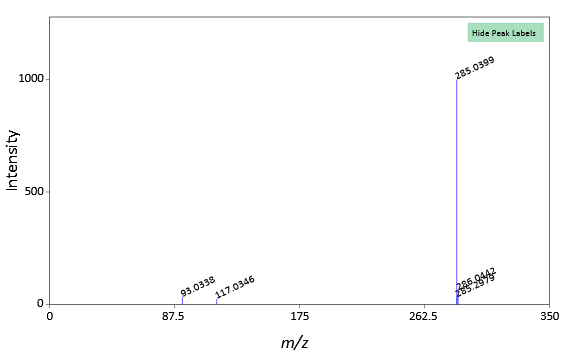


MS2：

285.0399、286.0442、117.0346

7. Aloeemodin


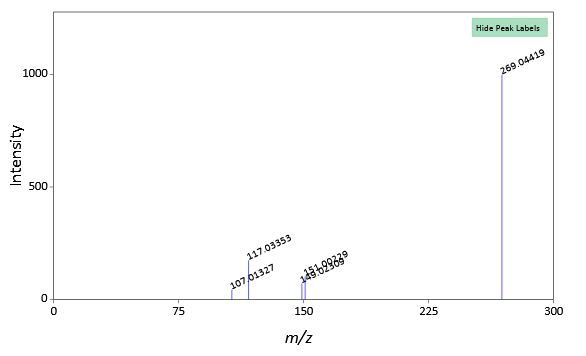


MS2：

117.03353、149.02309、269.04419

8. paeonoside


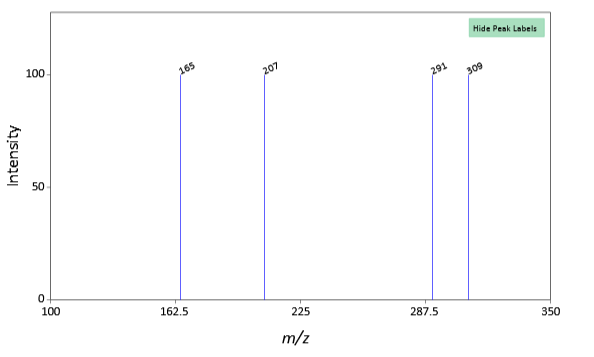


MS2：

309 、207、291

9. Linolenic acid

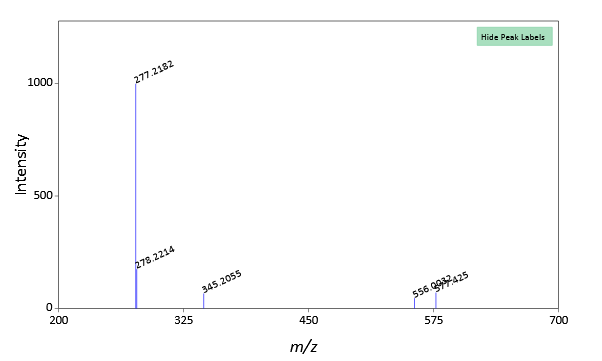


MS2：

277.2182、278.2214

10. Linoleic acid

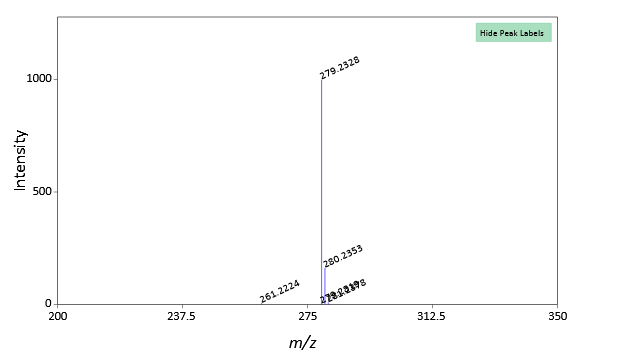


MS2：

279.2328、280.2353
