# Supplementary material for: Integrative network pharmacology, metabolomics and gut flora studies reveal mechanisms of action of Rhododendron molle (Blume) G. Don to ameliorate liver injury
Source: Front Microbiol. 2025 Sep 1;16:1570229. doi: 10.3389/fmicb.2025.1570229 (PMC12434124; doi:10.3389/fmicb.2025.1570229)
Supplement: Supplementary file 2 [file Supplementary_file_2.docx]

1.
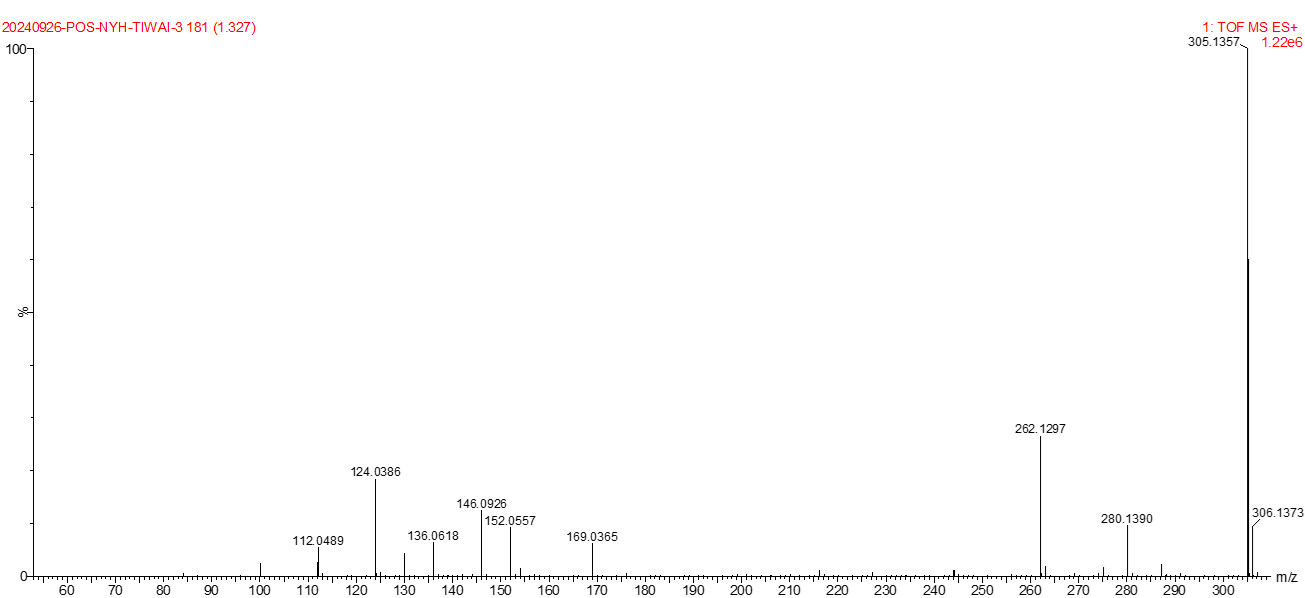
Resacetophenone


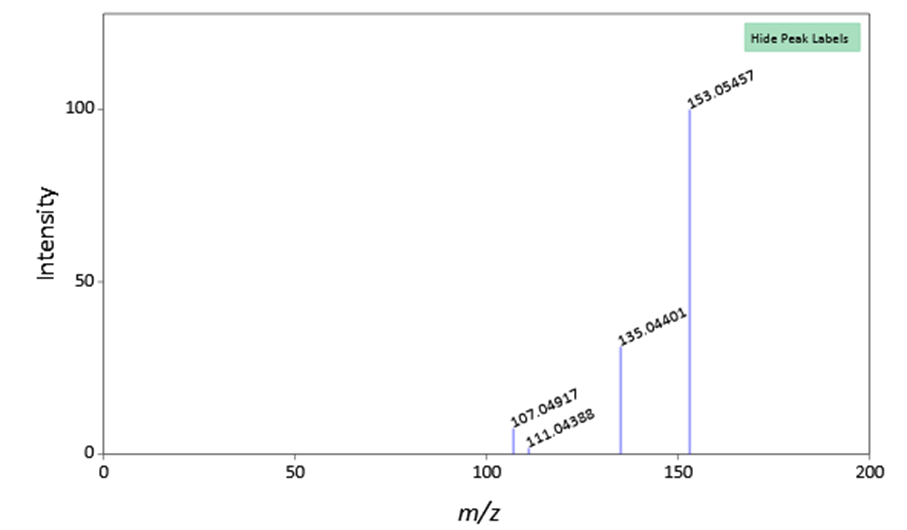


MS2：

112.0489、136.0618、152.0557

2. Laccaic Acid D

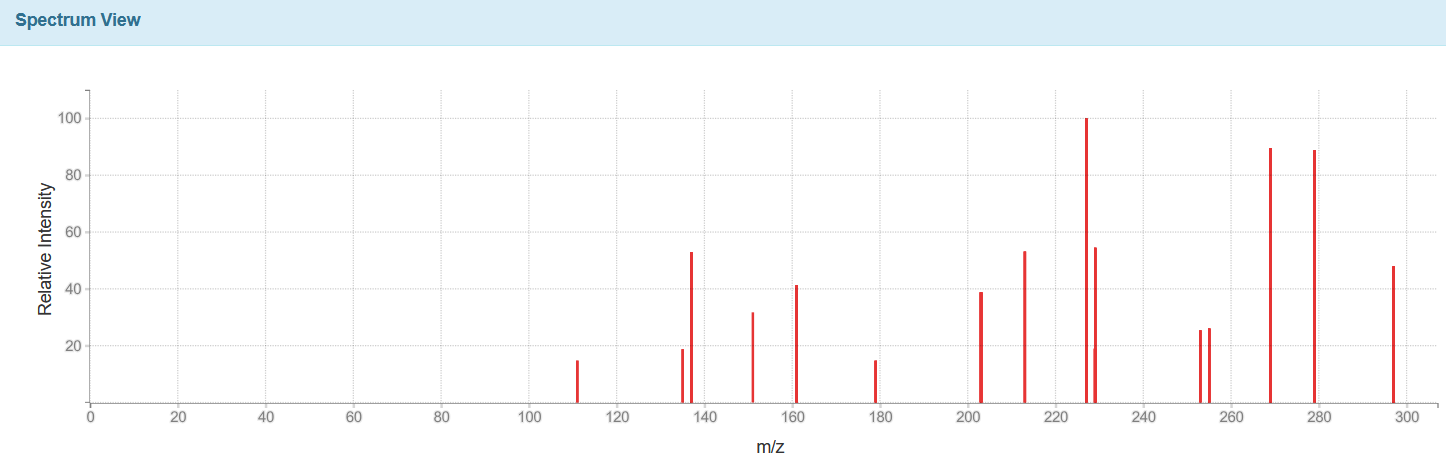


MS2：

269.1898、279.1747、297.1855

3.Quercetin

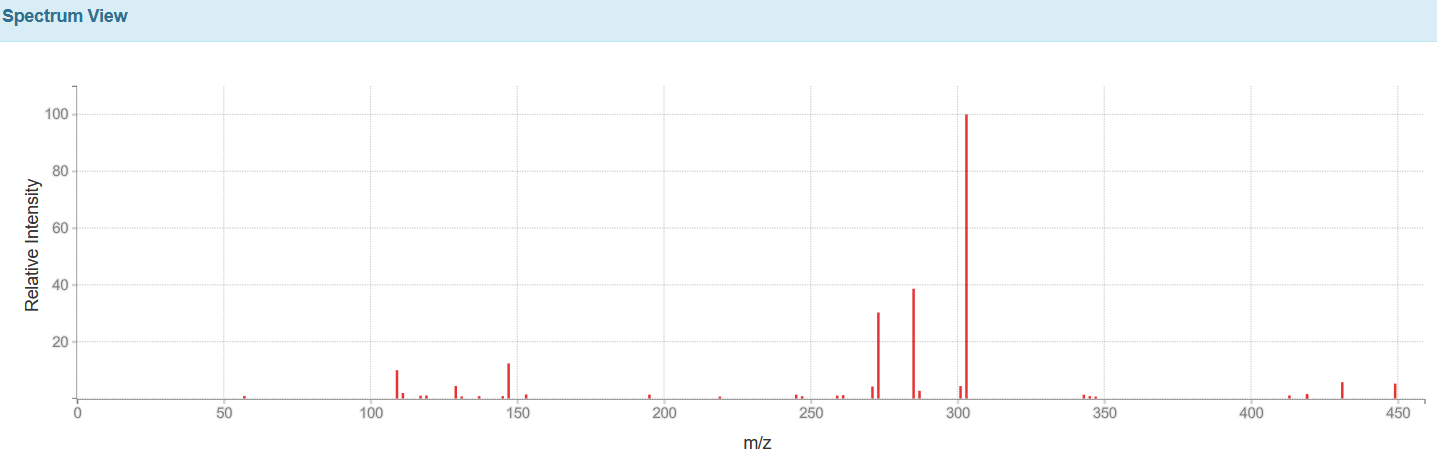


MS2：

303.0159、287.0556

4. Emodin

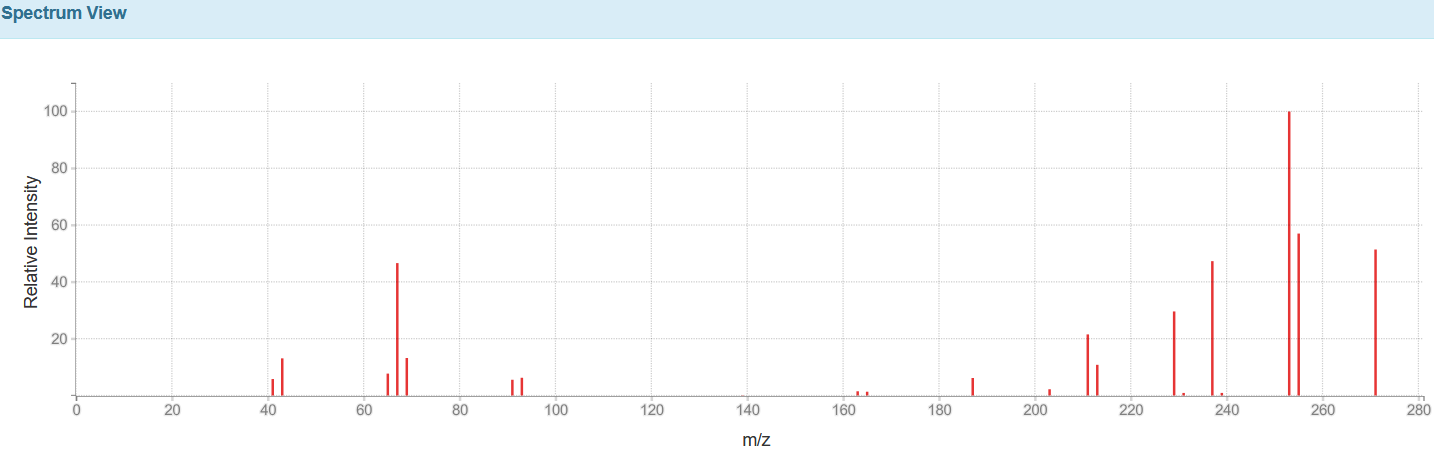


MS2：

163.0359、271.0606

5. Linolenic acid


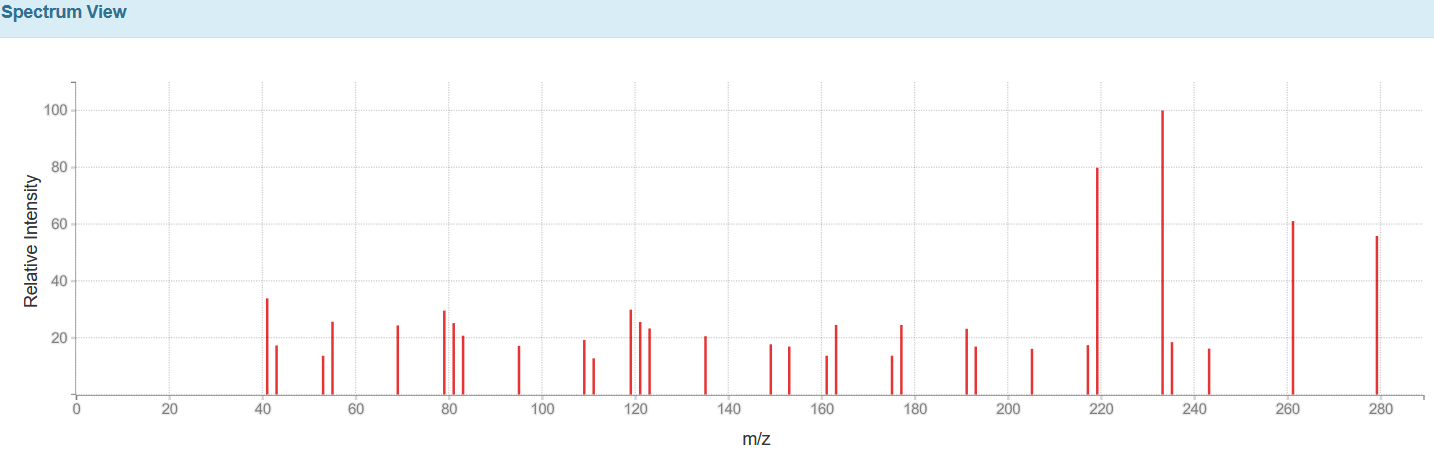


MS2：

243.2113、261.2218

6. sugiol

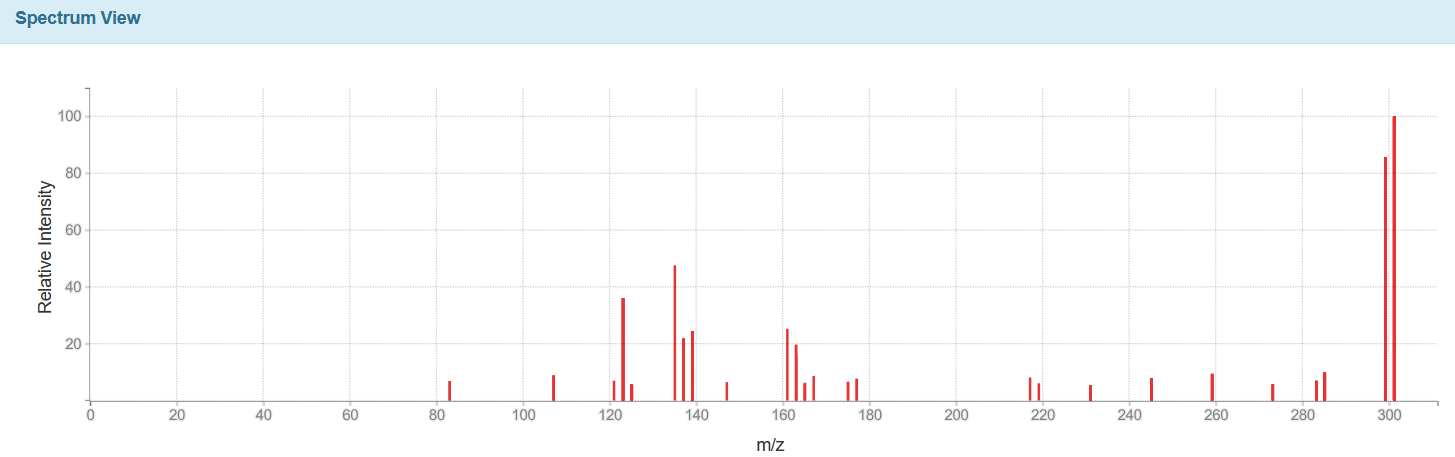


MS2：

139.1491、273.1849、123.1168

7．Benzylideneacetone


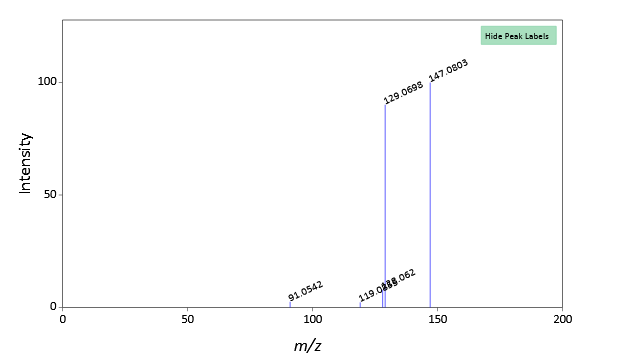


MS2：

147.0803、119.0855

8. Adenosine


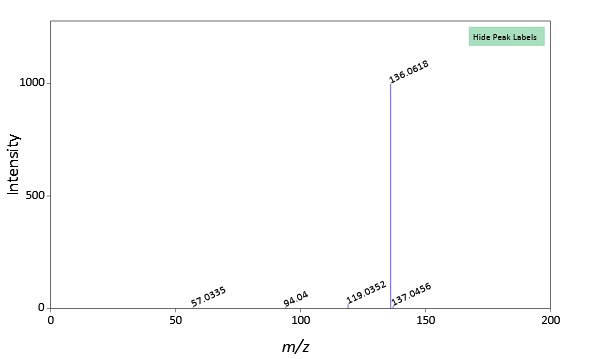


MS2：

136.0618 、119.0352 、137.0456
